# Exosomal miRNAs as circulating biomarkers for prediction of development of haematogenous metastasis after surgery for stage II/III gastric cancer

**DOI:** 10.1111/jcmm.15253

**Published:** 2020-05-08

**Authors:** Xin Liu, Kent‐Man Chu

**Affiliations:** ^1^ Department of Surgery The University of Hong Kong Hong Kong Hong Kong; ^2^ Department of Surgery The University of Hong Kong Queen Mary Hospital Hong Kong Hong Kong

**Keywords:** circulating biomarker, exosomal miRNA, gastric cancer, haematogenous metastasis

## Abstract

Exosomes secreted by living cancer cells can regulate metastasis. Exosomal miRNAs can reflect pathological conditions of the original cancer cells. Therefore, we aim to identify exosomal miRNAs as circulating biomarkers for haematogenous metastasis of gastric cancer. Pre‐treatment serum samples of eighty‐nine patients with stage II/III gastric cancer were collected. Thirty‐four of them developed haematogenous metastasis after surgery and the other fifty‐five did not. Extraction of exosomes was validated by western blot, transmission electron microscopy and nanoparticle tracking analysis. MiRNA qPCR array was performed in three matched pairs of samples. Internal control was selected from PCR array and validated in the remaining samples. Expressions of exosomal miRNAs were evaluated in the remaining samples by RT‐qPCR, as well as in gastric cancer tissue samples and cell culture medium. Expression levels of exosomal miRNAs were analysed with clinical characteristics. The results indicated thirteen up‐regulated and six down‐regulated miRNAs were found after normalization. MiR‐379‐5p and miR‐410‐3p were significantly up‐regulated in metastatic patients (*P* < .01). Higher expression of exosomal miR‐379‐5p or miR‐410‐3p showed shorter progression‐free survival of the patients (*P* < .05). It was also found that miR‐379‐5p and miR‐410‐3p were down‐regulated in gastric cancer tissue samples, while they were significantly up‐regulated in gastric cancer cell culture medium compared with cancer cells. In conclusion, exosomal miRNAs are promising circulating biomarkers for prediction of development of haematogenous metastasis after surgery for stage II/III gastric cancer.

## 1 INTRODUCTION

Gastric cancer is the second leading cause of cancer‐associated deaths worldwide.^1^ More than 70% of the global burden of gastric cancer cases concentrated in East Asia. Currently, surgical resection with or without adjuvant chemotherapy is the main treatment for patients without systemic metastasis on presentation. Nevertheless, despite a potentially curative surgery, about 40% of patients with advanced cancer may develop distant metastasis in the sequent 5 years.

Currently, most of these metastatic patients are diagnosed on clinical examination and/or diagnostic images. Therapy for such patients is limited, making the prognosis of patients with metastatic recurrence very poor.^2^ It is therefore important to identify biomarkers which could predict the subsequent development of metastatic disease. The ability to do so may help select patients at high risk of developing metastasis for aggressive neoadjuvant therapy before surgery or adjuvant therapy after surgery. In addition, such patients should be followed more closely after surgery.

Studies have indicated that metastasis is an early event in cancer development. Primary tumours create a favourable microenvironment in secondary organs and tissue sites for subsequent metastases.^3^ This is called the “seed” (the pre‐metastatic niche) and “soil” (secondary sites) theory. To transfer the “seed” to its appropriate “soil”, primary tumours secret extracellular vesicles.^4^ Exosomes are an important type of these vesicles. Intriguingly, exosomes released from primary cancer cells have a distinct genetic and epigenetic makeup, which allows them to undertake their tumorigenic function. These unique cancer‐specific fingerprints present in exosomes can be detected in circulation.^5^ Therefore, molecules in exosomes are promising circulating biomarkers for predicting metastasis of cancer, even before the metastasis could be detected clinically.

Recently, attention has been paid to exosomes as functional transporters in various pathological conditions including cancer.^6^, ^7^, ^8^ Importantly, molecules contained in exosomes can be transferred into cells of secondary sites, where these molecules affect gene expressions of the target cells, further to alter cell functions.^9^ Hence, exosomes play significant roles in intercellular communication to promote cancer progression.^10^ Exosomes range from 30 to 150 nm in diameter. They have been found in many body fluids including blood, saliva, urine, amniotic fluid and bile.^11^ Besides, deep‐sequencing experiments have shown that exosomes contain a very diverse RNA cargo.^12^ Most of the RNA cargo is 20‐200 nucleotides in length, including full‐length molecules such as miRNAs.^13^, ^14^

Exosomes are involved in the pathogenesis of cancer.^15^ For gastric cancer, studies indicated that exosomes were released by gastric cancer cells to perform certain functions in extracellular environment. For instance, it has been reported that oncogenic let‐7 miRNA family is abundant in the culture medium of a metastatic gastric cancer cell line AZ‐P7a.^16^ Another study suggested that exosomes derived from culture medium of gastric cancer cells stimulated the activation of NF‐κB pathway in macrophages to promote cancer progression.^17^ Exosomes extracted from gastric cancer cell culture medium promote the expression of adhesion molecules in mesothelial cells, which might play a critical role in the development of peritoneal metastasis of gastric cancer.^18^ Three studies used exosomal molecules as potential biomarkers for the pathological status of gastric cancer.^19^, ^20^, ^21^ However, these exosomal molecules were of large size and none of these studies concerned the detection of gastric cancer metastasis.

Studies have shown that dysregulation of miRNAs was involved in initiation and progression of various malignancies, including gastric cancer.^22^, ^23^ MiRNAs belong to a group of small non‐coding RNAs around 18‐25 nucleotides in length. They bind to complementary sequences in the 3′‐untranslated regions (3′‐UTR) of target mRNAs to induce degradation or translational repression.^24^, ^25^ MiRNAs are tissue specific and even cells specific within those tissues. They are potentially useful for diagnosis, predicting clinical outcome or acting as therapeutic targets in patients with cancer. The unique pattern of miRNAs in gastric cancer provides the possibility of applying miRNAs as biomarkers for monitoring gastric cancer.

MiRNAs are suitable for circulating biomarkers, as (a) the relatively smaller number of candidate sequences and thus relative ease of analysis and (b) stability due to small size (18‐25 nt) and hence robustness of detection. In addition, miRNAs from whole serum or plasma may include endogenous cellular miRNAs derived from apoptotic or necrotic cells. Serum/plasma miRNAs, which are not associated with vesicles, were reported to show differential stability to treatment by RNase. Hence, the unique nature of the exosomal bilayer allows miRNA to be protected from degradation, making it an ideal source for biomarkers.

In this study, we compared the expression profiles of exosomal miRNAs in the preoperative serum of the patients who developed haematogenous metastasis to those without metastasis in at least 5‐year follow‐up period. Dysregulated serum exosomal miRNAs from profiling were evaluated in another set of serum samples. Expressions of exosomal miRNAs were studied for the associated clinical characteristics and progression‐free survival. Expressions were also evaluated in gastric cancer tissue samples and cell line culture medium.

This is the first study to identify novel circulating exosomal miRNAs for prediction of development of haematogenous metastasis of gastric cancer. Serum exosomal miRNAs may act as circulating biomarkers for evaluation of metastatic status of patients with gastric cancer. It will be helpful for development of cost‐effective and mini‐invasive circulating biomarkers for monitoring gastric cancer.

## 2 MATERIALS AND METHODS

### 2.1 Human samples

The collection and storage of samples had been approved by the Ethics Committee of The University of Hong Kong. This project was performed in accordance with relevant guidelines/regulations. All of the samples were obtained with participants’ informed consent.

Human gastric cancer serum samples and paired tumour/adjacent non‐tumour tissue samples were collected from the patients with gastric cancer in Queen Mary Hospital, Hong Kong. None of the patients received preoperative treatment. All samples were immediately processed and stored at −70°C. Blood will be centrifuged at 1600 *g* for 10 minutes at 4°C, and serum will be transferred to new tubes followed by further centrifugation at 16 000 *g* for 10 minutes at 4°C. The supernatant will be transferred to new tubes. From this process, only the serum but not the blood cells, dead cells or cell debris will be collected for following study.

### 2.2 Gastric cancer cell lines

Human gastric cancer cell lines AGS and SNU1 (ATCC) were used in this study. Both cell lines were established from gastric cancer tissue samples of original site (stomach). Cells were cultivated in RPMI1640 medium (Gibco BRL) supplemented with 10% exosome‐depleted foetal bovine serum (FBS; SBI, System Biosciences). All cells were incubated at 37°C in a humidified incubator which contains 5% CO2.

### 2.3 Extraction of exosomes and exosomal miRNAs

Exosomes were extracted from serum samples using the ExoQuick Exosome Isolation Kit (SBI, System Biosciences). 500 µL serum samples were incubated with 120 µL ExoQuick™ for 60 minutes at 4°C. Then, the ExoQuick™‐serum samples were centrifuged twice at 800 *g* for 30 and 5 minutes, respectively, in order to remove the supernatant. The pellet was resuspended in PBS.

Exosomal RNAs were extracted using the SeraMir exosome RNA kit (SBI) according to the manufacturer's instructions. The quality and quantity of the RNAs were measured by NanoDrop 1000. The same amount of serum samples was applied for exosomal miRNAs extraction. And the same amount of miRNAs according to NanoDrop concentration was applied for reverse transcription (RT). So the measurements among different groups were comparable.

### 2.4 Western blot

In this study, CD9 and HSP70 were markers for validation of exosomes. Western blot was performed as previous described.^26^ Briefly, exosomes were extracted and lysed by RIPA Buffer (Sigma Chemical Co.). The concentration of proteins in each sample was evaluated by BCA Protein Assay. This assay produces a linear response curve, which allows accurate determination of unknown protein concentrations and provides a high dynamic range. With concentration of each sample, equal amount of protein was used for Western blot. Samples containing equal amount of protein were separated by SDS‐PAGE and electro blotted onto Immobilon‐P Transfer Membrane (Applied Biosystems). The membrane was blocked with 5% no‐fat milk, followed by incubation with antibodies specific for anti‐CD9 and anti‐HSP70 (1:1000; SBI) and anti‐β‐actin (1:20 000; Cell Signaling Technology), respectively. Blots were then incubated with anti‐rabbit or antimouse secondary antibody conjugated to horseradish peroxidase (Amersham Pharmacia) accordingly. The signals were captured by FUJI Medical X‐Ray Film and developed by the FUJI system.

### 2.5 Transmission electronic microscopy

Exosomes were visualized using transmission electron microscopy (TEM) to validate their morphology and general size, according to the method described before.^27^ Generally, exosome suspension was fixed in 2% paraformaldehyde. A small amount of this mix was transferred onto each of 2 formvar‐carbon‐coated electron microscopy grids. PBS was placed on a sheet of parafilm and grids transferred with the sample membrane side facing down using clean forceps. The grids were kept wet on the side of the membrane during all steps, but dry on the opposite side. The grids were transferred to 1% glutaraldehyde before transferring to distilled water. This was repeated several times. To contrast the samples, grids were transferred to uranyl‐oxalate solution pH7, before transferring to methyl‐cellulose‐UA, placing the grids on a glass dish covered with parafilm on ice. The grids were removed with stainless steel loops and excess fluid blotted gently on Whatman no.1 filter paper. Grids were left to dry and stored in appropriate grid storage boxes. Grids were observed with Thermo Fisher Tecnai transmission electron microscope.

### 2.6 Nanoparticle tracking analysis

Nanoparticle tracking analysis (NTA) was performed to quantify exosomes in serum samples.^28^ The number and size of exosomes were directly tracked using the NanoSight NS 300 system (NanoSight Technology). Exosomes were resuspended in PBS at a concentration of 5 μg/mL, then diluted 100‐ to 500‐fold, to achieve between 20 and 200 objects per frame. Samples were manually injected into the sample chamber at ambient temperature. Each sample was configured with a 488 nm laser and a high‐sensitivity scientific complementary metal‐oxide‐semiconductor (sCMOS) camera and was measured in triplicate at camera setting 13 with an acquisition time of 30 seconds and a detection threshold setting of 7. At least 100 completed tracks will be analysed per video. Finally, data were analysed using the NTA analytical software (version 2.3).

### 2.7 miRCURY LNA™ miRNA miRNome PCR Array

MiRCURY LNA™ miRNA miRNome PCR panels were applied for miRNA profiling of exosomal miRNAs from three pairs of matched metastatic and non‐metastatic serum samples. The panels contained 752 miRNAs for profiling for each sample. The matched samples were with the same stage, race, gender, similar age and no previous malignancy, in order to eliminate variations of physical conditions. Expressions of 752 miRNAs were evaluated in each sample (N = 6, 3 matched pairs) with PCR array. Expressions of the miRNAs were compared in each pair, two pairs and three pairs of the samples.

### 2.8 Reverse transcription‐quantitative polymerase chain reaction

Total exosomal miRNAs were reverse transcribed to cDNA using miRCURY LNA™ RT Kit (Exiqon) according to the manufacturer's instructions. Quantitative PCR was performed using miRCURY LNA™ SYBR Green Mix (Exiqon) in Vii7A real‐time PCR system (Applied Biosystems). The miRNA‐specific primer sequences were provided by Exiqon based on the miRNA sequences obtained from the miRBase database. At the end of the PCR cycles, melting curve analyses were performed. Fold changes in expression of each miRNA were calculated by a comparative threshold cycle (*C*_t_) method using the formula: $2^{-[\Delta C_{t}\left(\text{Internal}\;\text{Control}\right)-\Delta C_{t}\left(\text{sample}\right)]}$ . Currently, there is no standard internal control for exosomal miRNAs. MiRNAs with relatively abundant and consistent expressions released from profiling were applied as candidate internal controls.

### 2.9 Statistics

Statistical analysis was carried out using Statistical Package for Social Sciences (SPSS) 24.0 for Windows (SPSS Inc). Mann‐Whitney test was applied for analysis of expression differences of exosomal miRNAs between two groups. Log‐rank test was applied for progression‐free survival analysis. Chi‐square test or Fisher's exact test was used to analyse clinic‐pathological parameters. Wilcoxon's sign‐rank test was applied for analysis of expression differences in paired tissue samples. Student's *t* test was used to analyse the results expressed as mean ± SD. Differences were considered significant when *P* ≤ .05.

## 3 RESULTS

### 3.1 Patient recruitment and study design

In our cohort of patients, none of the patients in stage I developed metastasis in 5‐year follow‐up after operation. Patients in stage IV already had distant metastasis when they were diagnosed. Therefore, patients in stage II and stage III were included in this study. Among the patients in stage II/III, Chinese patients were included to eliminate racial differences. Only the patients with pre‐treatment serum samples were included. Finally, 89 patients were included. All these patients had been followed up for at least 5 years (or until the date of death for metastatic patients). Definition of haematogenous metastasis in this study included distant metastasis to liver, lung, bone, brain, or skin and soft tissue (excluding peritoneum). The diagnostic criteria of haematogenous metastasis were stated in Table S1. Patients who developed metastasis within 5 years after operation were categorized as metastatic patients, while patients without metastasis during 5‐year follow‐up were categorized as non‐metastatic ones. Among these patients, 34 of them developed haematogenous metastasis within 5 years after operation (Figure S1).

The characteristics of these patients, including age, gender, comorbidity, presenting symptoms and signs, operative findings and staging had been collected into our standard electronic database. These serum samples were subjected to miRNA profiling and RT‐qPCR. Analysis of clinical characteristics of the patients with metastasis and without metastasis was indicated in Table 1. The results suggested there was no significant difference between these two groups in gender and age. The difference in stages among II, IIIA and IIIB was consistent with higher risk of metastasis in more advanced stages. The samples were then applied for following study.

**Table 1:** Clinical characteristics of the patients with metastasis vs no metastasis

	Metastasis	No metastasis	*P*‐value
Gender			
Male	19	36	.3798
Female	15	19	
Age (y)			
Mean	67.5	61.9	.0846
Median	69 (32‐87)	64 (25‐84)	
Stage			
II	11	22	.5060
III	23	33	
Stage			
II	11	22	.0449
IIIA	8	22	
IIIB	15	11	

Metastasis on follow‐up (N = 34) vs no metastasis (N = 55).

### 3.2 Validation of extraction of exosomes

**Figure 1:**
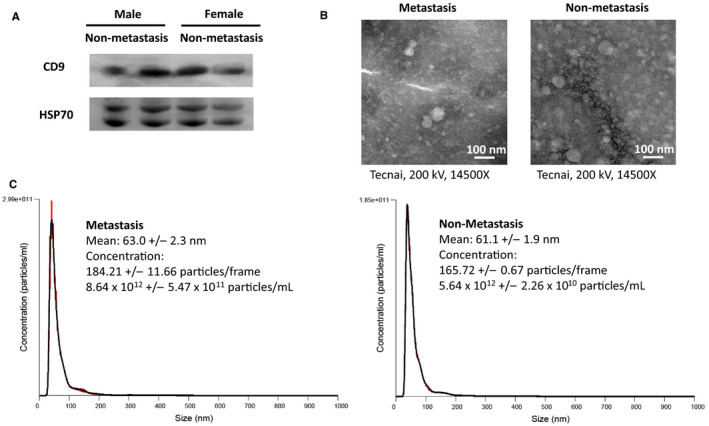
Validation of extraction of exosomes in serum samples. A, Western blot analysis indicated the expressions of exosomal surface biomarkers CD9 and HSP70 in metastasis and non‐metastasis serum samples. B, Morphology and general size of exosomes visualized by transmission electron microscopy (TEM) under 200 kV and 14 500 times enlargement. C, Quantitation and distribution of extracellular vesicles by nanoparticle tracking analysis (NTA). The result showed that the mean sizes of the vesicles were 63.0 ± 2.3 nm for metastasis sample and 61.1 ± 1.9 nm for non‐metastasis sample. The concentrations were 8.64 × 10^12^ ± 5.47 × 10^11^ particles/mL for metastasis sample and 5.64 × 10^12^ ± 2.26 × 10^10^ particles/mL for non‐metastasis sample. The mean size and the peak indicated that the extracellular vesicles from extraction were mostly exosomes

To confirm the extracellular vesicles were mostly exosomes, Western blot was performed to evaluate the surface biomarkers of the vesicles. Western blot analysis indicated the expressions of surface biomarkers CD9 and HSP70 in both metastasis and non‐metastasis serum samples Figure 1A. Transmission electron microscopy (TEM) was also applied to visualize morphology and general size of the vesicles. The result indicated the vesicles were circle‐like sharp, consistent with the morphology of exosomes. The diameters of the vesicles were generally less than 100 nm, within the size of exosomes (30‐150 nm) Figure 1B. In addition, nanoparticle tracking analysis was performed to analyse the quantitation and distribution of the vesicles. The result showed that the mean sizes of the vesicles were 63.0 ± 2.3 nm for metastasis sample and 61.1 ± 1.9 nm for non‐metastasis sample. These sizes were consistent with those observed under transmission electron microscopy. The concentrations were 8.64 × 10^12^ ± 5.47 × 10^11^ particles/mL for metastasis sample and 5.64 × 10^12^ ± 2.26 × 10^10^ particles/mL for non‐metastasis sample Figure 1C. The above results validated that the extracellular vesicles from extraction were mostly exosomes.

### 3.3 Exosomal miRNAs profiling in three pairs of serum samples

**Figure 2:**
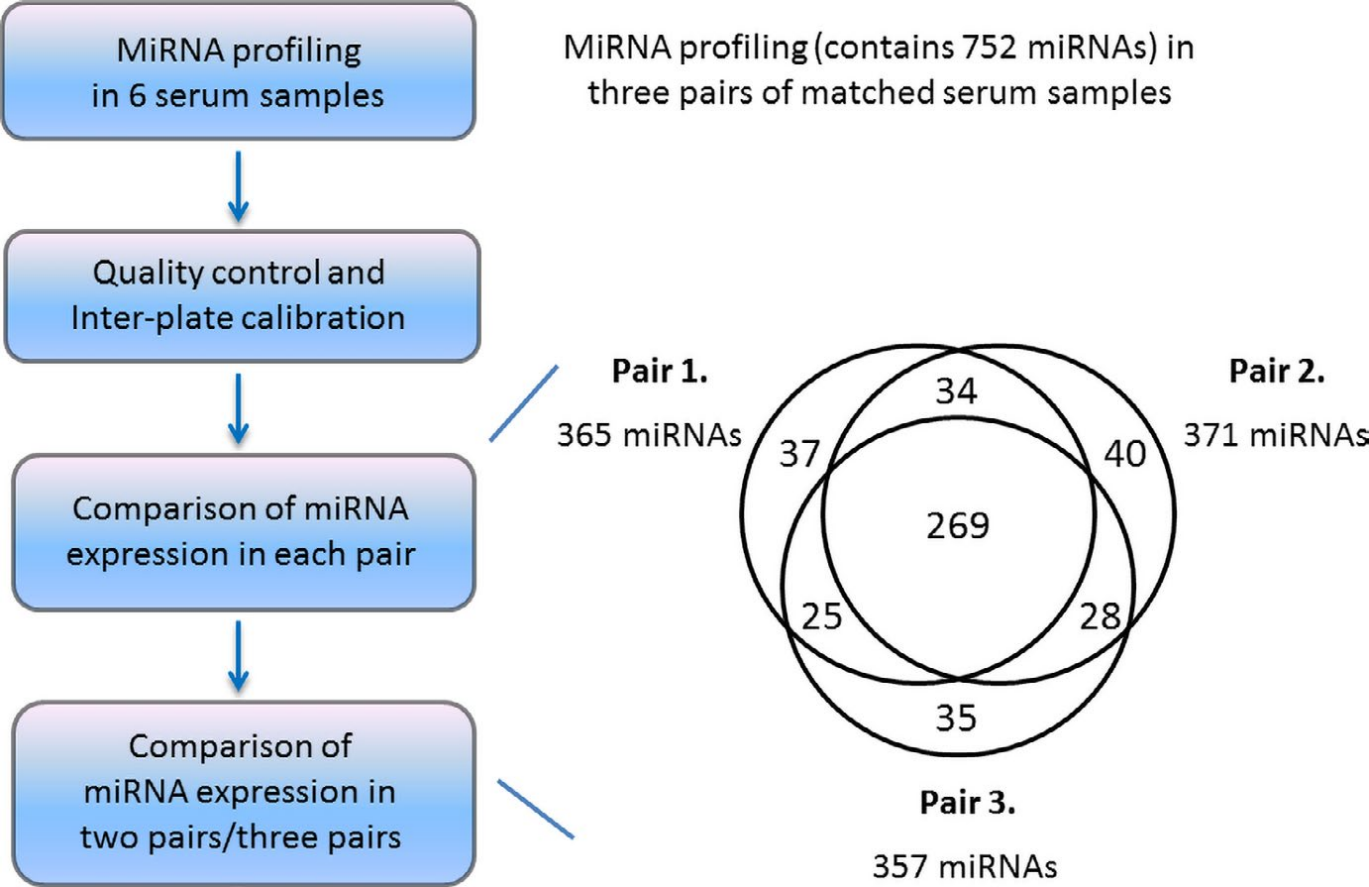
Analysis of miRCURY LNA™ miRNA miRNome PCR Array. General workflow of miRNA profiling was indicated on the left side. Quality control includes evaluation of quality of extraction with UniSp2, UniSp4 and UniSp 5, quality of reverse transcription with UniSp6 and Cel‐miR‐39‐5p, and inter‐plate calibration with UniSp3, triplicated. The numbers on the right side indicated the numbers of dysregulated miRNAs of the samples. There were 269 common dysregulated miRNAs among three pairs of samples

To identify dysregulated exosomal miRNAs in metastatic patients, three pairs of matched serum samples were applied for miRCURY LNA™ miRNA miRNome PCR Array. In order to eliminate physical variations, the matched samples were with same stage, race, gender, similar age and no previous malignancy Table 2. Both patients in each pair received surgical operation and the same type of adjuvant therapy after surgery. The quality of extraction of exosomal miRNAs was evaluated by spiked‐in small RNAs UniSp2, UniSp4 and UniSp5. The quality of reverse transcription (RT) efficiency of the samples was evaluated by spiked‐in small RNAs UniSp6 and Cel‐miR‐39‐5p. And UniSp3 (triplicated) was applied for inter‐plate calibrator. Expressions of 752 miRNAs were evaluated in each sample (N = 6, 3 matched pairs; Table S2). The expression of every miRNA was presented as a *C*_t_ value. If the *C*_t_ value was larger than 40 (*C*_t_ > 40), it was presented as “undetermined”. If expressions of a miRNA in both samples of a pair were “undetermined”, the miRNA in this pair would be excluded as the expressions were too low to be analysed. Therefore, there were 365 miRNAs for Pair 1, 371 miRNAs for Pair 2 and 357 miRNAs for Pair 3, with miRNA expression (*C*_t_ < 40). But miRNAs with expressions in each pair were not the same. Two hundred and sixty‐nine were the numbers of miRNAs with expressions (*C*_t_ < 40) in all three pairs of samples Figure 2.

**Table 2:** Clinical characteristics of three pairs of patients for miRNA profiling

Pair	Race	Gender	Age	Previous malignancy	TNM	Metastasis
1	Chinese	Male	69	No	II	Yes
	Chinese	Male	69	No	II	No
2	Chinese	Female	81	No	IIIA	Yes
	Chinese	Female	84	No	IIIA	No
3	Chinese	Female	48	No	IIIB	Yes
	Chinese	Female	51	No	IIIB	No

### 3.4 Normalization of miRNA expressions

**Figure 3:**
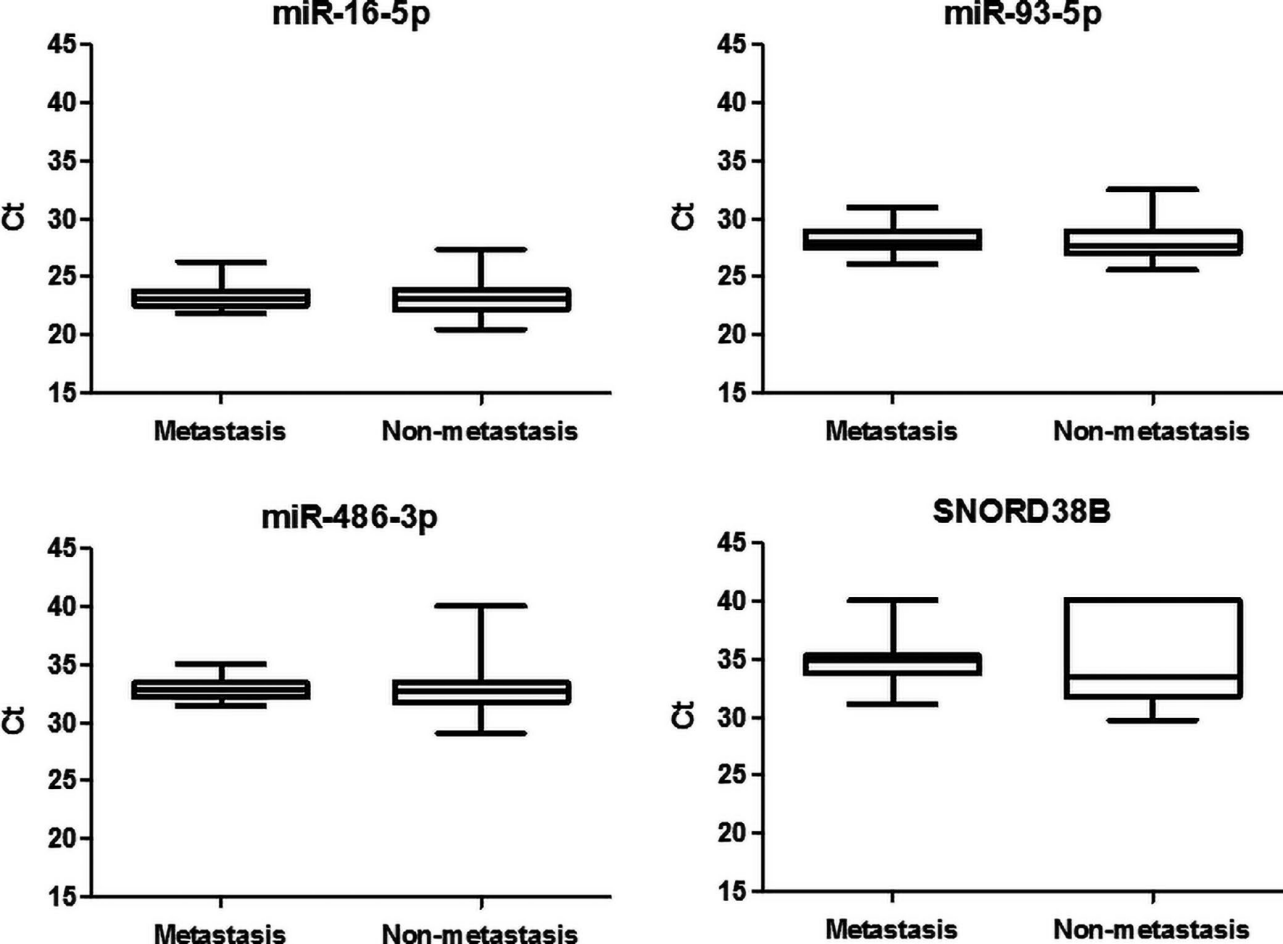
Expressions of candidate internal control small RNAs in the cohort. Expressions of candidate internal control small RNAs miR‐16‐5p, miR‐93‐5p, miR‐486‐3p and SNORD38B were shown. MiR‐16‐5p and miR‐93‐5p were chosen for internal controls in this study for their relatively abundant and consistent in our cohort of serum samples

To clarify whether the dysregulated miRNAs were up‐regulated or down‐regulated, internal control was needed to normalize the expressions. Currently, there is no standard internal control for exosomal miRNAs. From the qPCR array, we found *C*_t_ values of miR‐16‐5p, miR‐93‐5p, miR‐486‐3p and SNORD38B were more consistent among the six samples. So we chose these four small RNAs for candidate internal controls. Expressions of these small RNAs were evaluated in the remaining serum samples. The result indicated that the coefficient of variations of miR‐16‐5p was 6.12% (mean = 23.258, SD = 1.424), and the coefficient of variations of miR‐93‐5p was 5.20% (mean = 28.194, SD = 1.466) in the validation set of samples (N = 31 for metastasis and N = 52 for non‐metastasis). But the expressions of miR‐486‐3p or SNORD38B were “undetermined” (*C*_t_ > 40) in some of the samples. It indicated the expressions of miR‐486‐3p or SNORD38B were too low in some of the samples to be analysed as an internal control. So we chose miR‐16‐5p and miR‐93‐5p as internal controls considering their relative abundance and consistent expressions among the samples Figure 3.

To double‐check the expressions of exosomal miRNAs, both miR‐16‐5p and miR‐93‐5p were selected to be internal controls in this study. With normalization to miR‐16‐5p, 36 miRNAs were up‐regulated and 7 miRNAs were down‐regulated in 269 commonly dysregulated miRNAs. With normalization to miR‐93‐5p, 23 miRNAs were up‐regulated and 27 miRNAs were down‐regulated (Table S3). Thirteen up‐regulated and six down‐regulated miRNAs were found to be overlapped in both normalization analysis Table 3.

**Table 3:** Average fold changes of miRNAs in metastatic patients comparing with non‐metastatic control

Targets	Average (miR‐16‐5p)	Average (miR‐93‐5p)
	M/NM	M/NM
Up‐regulation		
hsa‐let‐7c‐5p	6.936	2.569
hsa‐let‐7d‐5p	4.521	2.360
hsa‐let‐7e‐5p	3.619	1.940
hsa‐miR‐144‐5p	4.656	2.802
hsa‐miR‐186‐5p	4.667	1.800
hsa‐miR‐20b‐5p	69.990	12.134
hsa‐miR‐331‐3p	32.978	20.656
hsa‐miR‐379‐5p	127.829	49.628
hsa‐miR‐410‐3p	321.130	103.873
hsa‐miR‐495‐3p	24.731	41.811
hsa‐miR‐532‐5p	3.466	1.727
hsa‐miR‐98‐5p	5.873	2.369
hsa‐miR‐99b‐5p	3.824	1.651
Down‐regulation		
hsa‐miR‐450a‐5p	0.516	0.347
hsa‐miR‐450b‐5p	0.006	0.005
hsa‐miR‐505‐5p	0.141	0.041
hsa‐miR‐589‐5p	0.242	0.316
hsa‐miR‐603	0.187	0.140
hsa‐miR‐934	0.346	0.230

### 3.5 RT‐qPCR in the remaining serum samples

The expressions of exosomal miRNAs were evaluated in the remaining serum samples. Seven of the dysregulated miRNAs (let‐7c‐5p, miR‐144‐5p, miR‐379‐5p, miR‐410‐3p, miR‐98‐5p, miR‐505‐5p and miR‐934) were chosen for further study according to their fold changes and association with cancer development (Table S4). Firstly, around half of the remaining samples (N = 18 for metastasis, N = 30 for non‐metastasis) were applied for evaluation by RT‐qPCR. The result suggested that there was no obvious difference of let‐7c‐5p, miR‐98‐5p or miR‐934 between two groups (Figure S2). Hence, four miRNAs, miR‐144‐5p, miR‐379‐5p, miR‐410‐3p and miR‐505‐5p, were applied for evaluation in the remaining of the samples. The results of RT‐qPCR of these four miRNAs indicated that there was no or consistent significant difference of miR‐144‐5p or miR‐505‐5p in the remaining samples with normalization of miR‐16‐5p and miR‐93‐5p (Figure S3).

**Figure 4:**
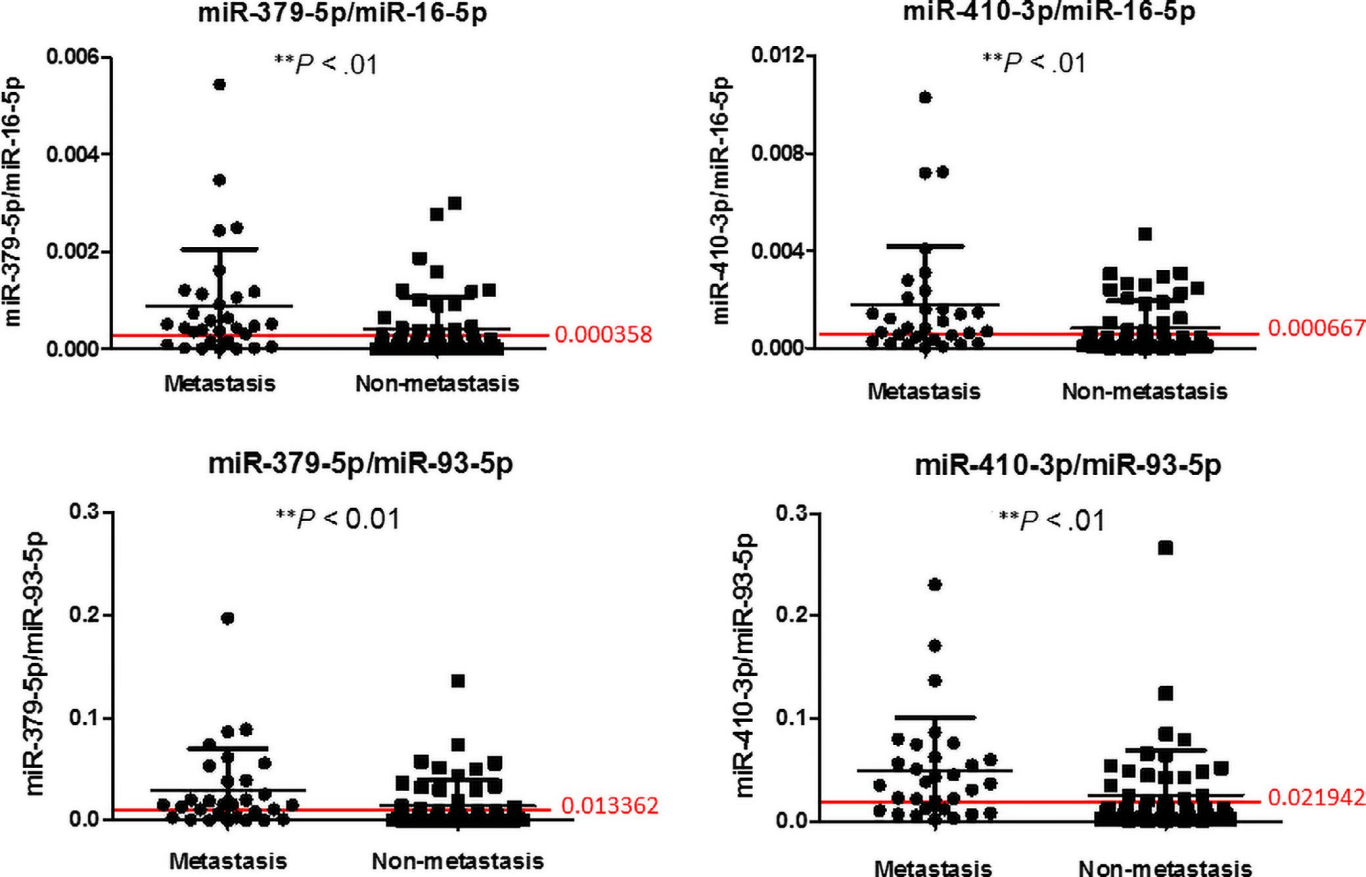
Expressions of exosomal miR‐379‐5p and miR‐410‐3p in serum samples. The expressions of exosomal miR‐379‐5p and miR‐410‐3p in serum samples of the metastasis group and non‐metastasis group were shown. The upper side was the expressions normalized with miR‐16‐5p. The lower side was the expressions normalized with miR‐93‐5p. Exosomal miR‐379‐5p and miR‐410‐3p were significantly up‐regulated in the metastasis group. N = 31 for metastasis, N = 52 for non‐metastasis, **P < .01. The bars and values in red indicated the optimal cut‐off points for these cohorts

RT‐qPCR indicated that exosomal miR‐379‐5p and miR‐410‐3p were significantly up‐regulated in the metastasis group (N = 31) comparing with the non‐metastasis group (N = 52; Figure 4, ^**^ *P* < .01). ROC method was applied for determination of optimal cut‐off points. The bars and values in red showed the optimal cut‐off points for these cohorts Figure 4.

### 3.6 Sensitivity, specificity and clinical characteristics

**Figure 5:**
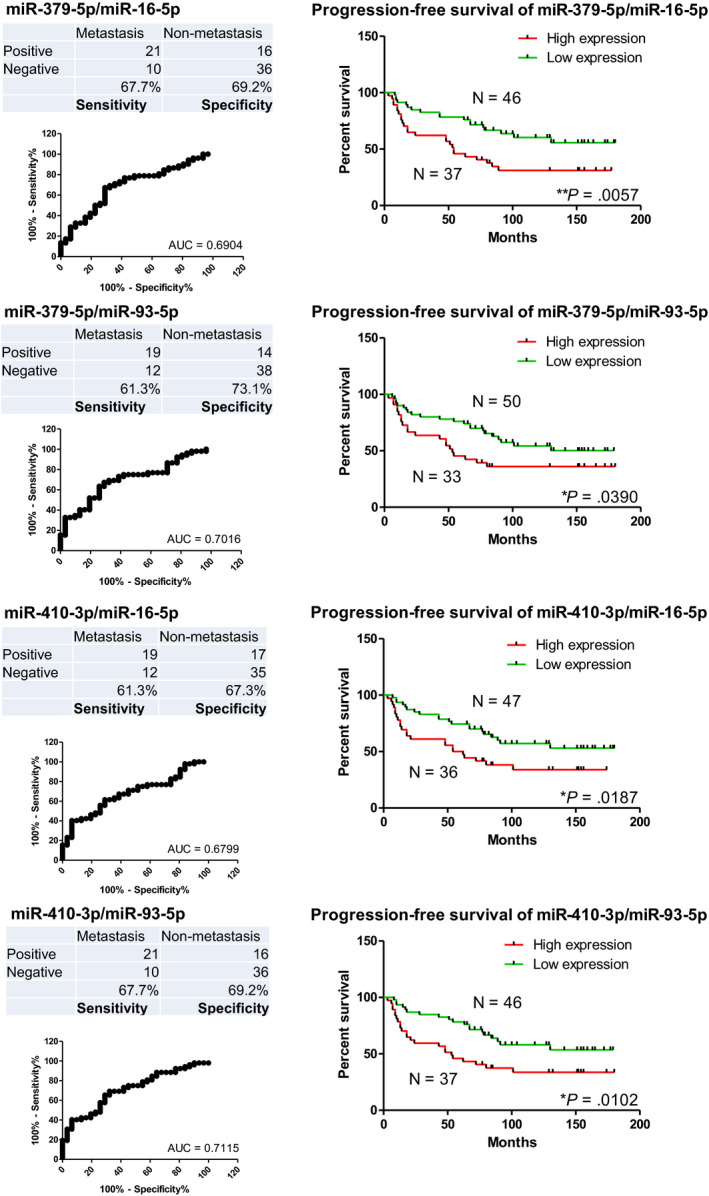
Sensitivity, specificity, AUC and progression‐free survival of exosomal miR‐379‐5p and miR‐410‐3p. Tables and figures on the left side indicated sensitivity, specificity and AUC of exosomal miR‐379‐5p and miR‐410‐3p in the cohort with the optimal cut‐off points. Figures on the right side indicated progression‐free survival of exosomal miR‐379‐5p and miR‐410‐3p for this cohort. Patients with higher expressions of serum exosomal miR‐379‐5p or miR‐410‐3p showed significantly worse progression‐free survival

With the above optimal cut‐off points, expression values larger than cut‐off points were categorized as positive, while expression values smaller than cut‐off points were categorized as negative. Sensitivity and specificity of exosomal miR‐379‐5p and miR‐410‐3p were indicated in Figure 5. Statistical analysis revealed the AUC were 0.6904 for miR‐379‐5p/miR‐16‐5p, 0.7016 for miR‐379‐5p/miR‐93‐5p, 0.6799 for miR‐410‐3p/miR‐16‐5p and 0.7115 for miR‐410‐3p/miR‐93‐5p (Figure 5, left side). In addition, the progression‐free survival of the patients with higher expression of exosomal miR‐379‐5p or miR‐410‐3p was significantly worse than the patients with lower expression (Figure 5, right side). This suggested that exosomal miR‐379‐5p and miR‐410‐3p were associated with gastric cancer progression.

Association of expressions of these two miRNAs and distant metastatic sites was summarized in Table 4. It indicated that higher expressions of exosomal miR‐379‐5p were associated with distant metastasis, especially to lung and bone, whereas higher expressions of exosomal miR‐410‐3p were associated with distant metastasis especially to liver and lung.

**Table 4:** Association of expression levers of miR‐379‐5p/miR‐410‐3p and distribution of metastatic sites

Metastatic site	Positive	Negative	Metastatic site	Positive	Negative
*miR‐379‐5p/miR‐16‐5p*			*miR‐379‐5p/miR‐93‐5p*		
Liver	9	8	Liver	7	10
Lung	10	2	Lung	9	3
Bone	4	1	Bone	5	0
Brain	2	0	Brain	1	1
Skin and soft tissue	1	1	Skin and soft tissue	1	1
*miR‐410‐3p/miR‐16‐5p*			*miR‐410‐3p/miR‐93‐5p*		
Liver	11	6	Liver	10	7
Lung	6	6	Lung	7	5
Bone	2	3	Bone	5	0
Brain	2	0	Brain	1	1
Skin and soft tissue	1	1	Skin and soft tissue	1	1

### 3.7 Expressions of miRNAs in gastric cancer tissue samples and cell culture medium

**Figure 6:**
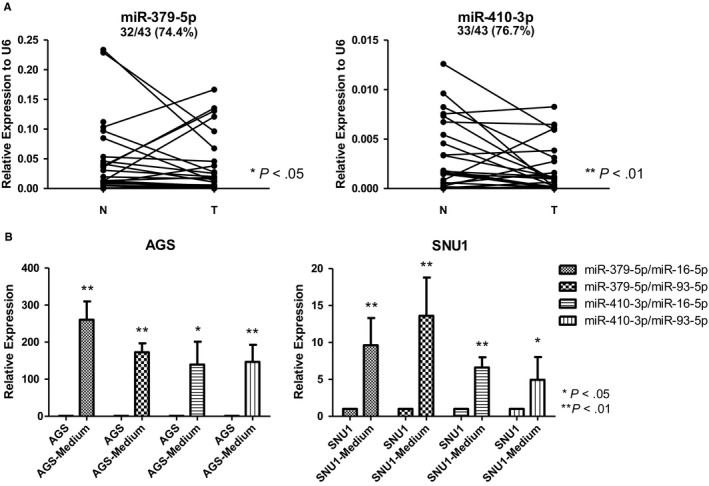
Expressions of miR‐379‐5p and miR‐410‐3p in gastric cancer tissue samples and cell culture medium. A, The expressions of miR‐379‐5p and miR‐410‐3p in gastric cancer pair tumour/adjacent non‐tumour tissue samples were shown. MiR‐379‐5p was down‐regulated in 32 out of 43 pairs (74.4%) of tissue samples. MiR‐410‐3p was down‐regulated in 33 out of 43 pairs (76.7%) of tissue samples. B, The expressions of miR‐379‐5p and miR‐410‐3p in gastric cancer cells and cell culture medium were shown. The cells were cultured with medium supplementary with exosome‐depleted FBS. It indicated that expressions of miR‐379‐5p and miR‐410‐3p in exosomes of cell culture medium were significantly higher than expressions of miR‐379‐5p and miR‐410‐3p in gastric cancer cells. *P < .05, **P < .01

To investigate the mechanism of higher expressions of exosomal miR‐379‐5p and miR‐410‐3p, the expressions of these two miRNAs were evaluated in gastric cancer paired tumour/ adjacent non‐tumour tissue samples (N = 43 pairs) by qPCR. The result indicated both miR‐379‐5p and miR‐410‐3p were significantly down‐regulated in gastric cancer tissue samples (*P < .05, 32 out of 43 pairs for miR‐379‐5p and **P < .01, 33 out of 43 pairs for miR‐410‐3p) Figure 6A. Expression of miR‐379‐5p or miR‐410‐3p was significantly down‐regulated in gastric cancer tissue samples, whereas exosomal miR‐379‐5p or miR‐410‐3p was up‐regulated in serum samples. We assumed gastric cancer cells secreted exosomes containing miR‐379‐5p/miR‐410‐3p to circulation.

To validate this assumption, expressions of miR‐379‐5p or miR‐410‐3p and exosomal miR‐379‐5p or miR‐410‐3p were evaluated in gastric cancer cell lines and relevant cell culture medium respectively. AGS and SNU1 were gastric cancer cell lines established from gastric cancer tissue samples (original site: stomach). The cells were culture with medium supplementary with exosome‐depleted FBS. The same amount of cDNA of cell line samples and serum samples were applied for qPCR. We found that both miR‐16‐5p and miR‐93‐5p were relatively abundant in cell line samples and serum samples. The Ct values of miR‐16‐5p or miR‐93‐5p were similar in cell line samples and serum samples. It suggested that miR‐16‐5p and miR‐93‐5p were highly exported in cell culture media from gastric cancer cells. The result showed that expressions of exosomal miR‐379‐5p and miR‐410‐3p in cancer cell culture medium were significantly higher than the expressions of miR‐379‐5p and miR‐410‐3p in gastric cancer cells Figure 6B. This suggested that exosomes might translocate miR‐379‐5p and miR‐410‐3p from cancer cells to medium.

## 4 DISCUSSION

Gastric cancer ranks as the second leading cause of cancer‐associated death worldwide. Metastasis is the fatal character of cancer. Today, patients who develop haematogenous metastasis after surgery are only diagnosed when they present with clinical signs and/or abnormality on imaging. Therefore, we investigated circulating biomarkers for prediction of subsequent metastasis for patients with stage II/III gastric cancer. Circulating biomarkers, referred to as “liquid biopsy”, are important for precision medicine. Advantages of liquid biopsy comparing with conventional biopsy include (a) it overcomes heterogeneity in tissue samples, (b) it is practical for serial sampling as it is mini‐invasive or non‐invasive and (c) it is sensitive and specific as each type of cancer contains its own genetic/epigenetic pattern.

By miRNA profiling and qPCR validation, we identify serum exosomal miR‐379‐5p and miR‐410‐3p as promising circulating biomarkers for prediction of development of haematogenous metastasis after surgery for stage II/III gastric cancer. Exosomal miR‐379‐5p and miR‐410‐3p significantly increased in metastatic patients comparing with non‐metastatic ones. Higher expressions of exosomal miR‐379‐5p or miR‐410‐3p were associated with worse progression‐free survival of the patients. These results indicated that exosomal miR‐379‐5p and miR‐410‐3p might contribute to gastric cancer progression.

We also found miR‐379‐5p and miR‐410‐3p were down‐regulated in gastric cancer tissue samples. As miRNAs could be selectively secreted from their original gastric cancer cells by exosomes, translocation of miR‐379‐5p and miR‐410‐3p from cancer cells to circulation might contribute to the down‐regulation of miR‐379‐5p and miR‐410‐3p in gastric cancer tissue samples. This assumption was validated in cell line model that expression levels of exosomal miR‐379‐5p or miR‐410‐3p in cell culture medium were much higher than their expression levels in gastric cancer cells.

Previous studies indicated that miR‐379‐5p was down‐regulated in lung cancer tissue samples and functioned as a tumour suppressor in lung cancer development.^29^, ^30^ Interestingly, another study indicated exosomal miR‐379‐5p was higher in plasma samples of lung cancer patients and exosomal miR‐379‐5p could be circulating biomarkers for lung cancer.^31^ These studies suggested that miR‐379‐5p, functioning as a tumour suppressor in lung cancer, could be secreted by exosomes from lung cancer cells to circulation, which is similar to ours. Our data also indicated that higher exosomal miR‐379‐5p was associated with gastric cancer metastasis to lung. It has been reported that miR‐379‐5p was down‐regulated and functioned as a tumour suppressor in various cancers, including gastric cancer.^32^ But exosomal miR‐379‐5p was increased in circulation. This suggested secretion of miR‐379‐5p by exosomes might be one of the mechanisms of down‐regulation of tumour suppressive miRNAs in carcinogenesis. However, the metabolism of exosomal miR‐379‐5p is needed to be further clarified as it seems miR‐379‐5p will not enter the distant cells to execute tumour suppressive roles.

Studies reported that miR‐410‐3p functioned as an oncogene in liver cancer, lung cancer and colorectal cancer,^33^, ^34^, ^35^ but as a tumour suppressor in osteosarcoma (bone), glioma (brain) and breast cancer.^36^, ^37^, ^38^ This evidence suggests that the roles of miR‐410‐3p are cancer‐specific and content dependent. The functions of miR‐410‐3p are complicated and miR‐410‐3p is a double‐sword in cancer initiation and progression.^39^ A study suggested miR‐410‐3p functioned as a tumour suppressor in gastric cancer.^40^ MiR‐410‐3p was also down‐regulated in gastric cancer tissue samples in our study. Secretion of miR‐410‐3p in exosomes from cancer cells to circulation might contribute to its down‐regulation in tissue samples. Exosomal miR‐410‐3p might enter into distant cells and play oncogenic roles in those cells. This might be one of the reasons for liver metastasis and/or lung metastasis of gastric cancer. But this could not explain gastric cancer metastasis to brain, bone or breast (soft tissue), as miR‐410‐3p executed as a tumour suppressor in these sites. It revealed that tumour metastasis is a complicated and comprehensive process. Other miRNAs and molecules (DNAs, long non‐coding RNAs, proteins, etc) were also involved in development of metastasis.

It has been reported exosomal miRNAs may be applied as circulating biomarkers for gastric cancer. For example, a study suggested that serum exosomal miR‐19b‐3p and miR‐106a‐5 could be potential biomarkers for detection of gastric cancer.^41^ Another study investigated six serum miRNAs as diagnostic biomarkers for gastric cancer and further indicated four serum exosomal miR‐10b‐5p, miR‐195‐5p, miR‐20a‐3p and miR‐296‐5p were significantly increased in patients with gastric cancer.^42^ Additionally, exosomal miRNA profiling in peritoneum lavage fluid showed that exosomal miR‐21 and miR‐1225‐5p might serve as prognostic biomarkers for peritoneal metastasis after curative gastric cancer resection.^43^

In this study, we focused on exosomal miRNAs as circulating biomarkers for prediction of development of haematogenous metastasis in stage II/III gastric cancer. We found exosomal miRNAs had been dysregulated in haematogenous metastatic gastric cancer patients, even before those patients presented with clinical signs and/or abnormal imaging. Exosomal miR‐379‐5p and miR‐410‐3p were significantly up‐regulated in the serum of gastric cancer patients who would develop haematogenous metastasis. Higher exosomal miR‐379‐5p or miR‐410‐3p was associated with worse progression‐free survival of the patients. This will be helpful for a selection of patients for aggressive neoadjuvant therapy before surgery, aggressive adjuvant therapy after surgery or closer follow‐up after surgery. In conclusion, serum exosomal miR‐379‐5p and miR‐410‐3p may be applied as circulating biomarkers for prediction of development of haematogenous metastasis after surgery for patients with stage II/III gastric cancer.

## CONFLICTS OF INTERESTS

The authors confirm that there are no conflicts of interest.

## AUTHOR CONTRIBUTIONS

Prof. KM Chu and Dr Michelle X. Liu designed this project. Prof. KM Chu collected the clinical samples. Dr Michelle X. Liu performed experiments and collected data. Dr Michelle X. Liu and Prof. KM Chu performed data analysis. Dr Michelle X. Liu and Prof. KM Chu wrote and revised the manuscript.

## Supporting information

Figures S1‐S3

Table S1

Table S2

Table S3

Table S4

Legends

## Data Availability

Supplementary Information accompanies this paper on *Journal of Cellular and Molecular Medicine* website.
