# Supplementary material for: Exosomal miRNAs as circulating biomarkers for prediction of development of haematogenous metastasis after surgery for stage II/III gastric cancer
Source: J Cell Mol Med. 2020 May 8;24(11):6220–32. doi: 10.1111/jcmm.15253 (PMC7294143; doi:10.1111/jcmm.15253)
Supplement: Supplementary file 1 — Figures S1‐S3 [file JCMM-24-6220-s001.docx]

**Supplementary Figures**

**Supplementary Figure 1. Subject recruitment for this study.** Chinese patients were recruited to eliminate racial differences in miRNA profiling. Patients with pre-treatment serum samples were recruited. Patients who developed haematogenous metastasis after gastrectomy were recruited as metastasis group. Patients who had completed 5-year follow-up period with no metastasis were recruited as non-metastasis group.

**Supplementary Figure 2. Expressions of exosomal let-7c-5p, miR-98-5p and miR-934 in serum samples.** Expressions of exosomal let-7c-5p, miR-98-5p and miR-934 in serum samples of the metastasis group and non-metastasis group were shown. *P* values were indicated on the figure. N=18 for metastasis, N=30 for non-metastasis.

**Supplementary Figure 3. Expressions of exosomal miR-144-5p and miR-505-5p in serum samples.** Expressions of exosomal miR-144-5p and miR-505-5p in serum samples of the metastasis group and non-metastasis group were shown. *P* values were indicated on the figure. N=31 for metastasis, N=52 for non-metastasis.

**Supplementary Tables**

Supplementary Table 1. Diagnostic criteria of haematogenous metastasis in this study.

Supplementary Table 2. List of miRNAs in the miRCURY LNA^TM^ miRNA miRNome PCR Panel.

Supplementary Table 3. Average fold changes of miRNAs in metastatic patients comparing with non-metastatic controls.

Supplementary Table 4. Primers of miRNAs in this study.
