# Supplementary material for: Exosomal miRNAs as circulating biomarkers for prediction of development of haematogenous metastasis after surgery for stage II/III gastric cancer
Source: J Cell Mol Med. 2020 May 8;24(11):6220–32. doi: 10.1111/jcmm.15253 (PMC7294143; doi:10.1111/jcmm.15253)
Supplement: Supplementary file 2 — Table S1 [file JCMM-24-6220-s002.docx]

Supplementary Table 1. Diagnostic criteria of haematogenous metastasis in this study.

| Metastatic Site | Diagnostic criteria |
| --- | --- |
| Liver | 1. Clinical, and/or 2. USG scan (Ultrasound scan), and/or 3. CT scan (Computed Tomography scan), and/or 4. FNA (Fine Needle Aspiration), and/or 5. Laparotomy. |
| Lung | 1. Clinical, and/or 2. CXR (Chest X-Ray), and/or 3. Sputum cytology, and/or 4. Pleural fluid cytology, and/or 5. Lesion FNA, and/or 6. CT thorax. |
| Bone | 1. Clinical, and/or 2. X-Ray, and/or 3. Bone scan, and/or 4. Skeletal survey, and/or 5. CT scan, and/or 6. MRI scan (Magnetic Resonance Imaging scan), and/or 7. Biopsy. |
| Brain | 1. Clinical, and/or 2. CT scan, and/or 3. MRI scan. |
| Skin and Soft Tissue | 1. Clinical, and/or 2. FNA, and/or 3. Incisional biopsy, and/or 4. Excisional biopsy. |
