# Supplementary material for: Exosomal miRNAs as circulating biomarkers for prediction of development of haematogenous metastasis after surgery for stage II/III gastric cancer
Source: J Cell Mol Med. 2020 May 8;24(11):6220–32. doi: 10.1111/jcmm.15253 (PMC7294143; doi:10.1111/jcmm.15253)
Supplement: Supplementary file 3 — Table S2 [file JCMM-24-6220-s003.docx]

Supplementary Table 2. List of miRNAs in the miRCURY LNA^TM^ miRNA miRNome PCR Panel.

| miRNA (human) | Order in 384  (for sorting by row) | Order in 384 (for sorting by column) | Panel plate position | microRNA target sequence | |
| --- | --- | --- | --- | --- | --- |
| **Panel I** |  |  |  | |  |
| hsa-miR-7-5p | 1 | 1 | A01 | | UGGAAGACUAGUGAUUUUGUUGU |
| hsa-miR-217 | 2 | 17 | A02 | | UACUGCAUCAGGAACUGAUUGGA |
| hsa-miR-337-5p | 3 | 33 | A03 | | GAACGGCUUCAUACAGGAGUU |
| hsa-miR-328-3p | 4 | 49 | A04 | | CUGGCCCUCUCUGCCCUUCCGU |
| hsa-miR-374b-3p | 5 | 65 | A05 | | CUUAGCAGGUUGUAUUAUCAUU |
| hsa-miR-143-3p | 6 | 81 | A06 | | UGAGAUGAAGCACUGUAGCUC |
| hsa-miR-623 | 7 | 97 | A07 | | AUCCCUUGCAGGGGCUGUUGGGU |
| hsa-miR-520c-3p | 8 | 113 | A08 | | AAAGUGCUUCCUUUUAGAGGGU |
| hsa-miR-557 | 9 | 129 | A09 | | GUUUGCACGGGUGGGCCUUGUCU |
| hsa-miR-218-5p | 10 | 145 | A10 | | UUGUGCUUGAUCUAACCAUGU |
| hsa-miR-136-5p | 11 | 161 | A11 | | ACUCCAUUUGUUUUGAUGAUGGA |
| hsa-miR-127-5p | 12 | 177 | A12 | | CUGAAGCUCAGAGGGCUCUGAU |
| hsa-miR-140-5p | 13 | 193 | A13 | | CAGUGGUUUUACCCUAUGGUAG |
| hsa-miR-31-3p | 14 | 209 | A14 | | UGCUAUGCCAACAUAUUGCCAU |
| hsa-miR-20b-3p | 15 | 225 | A15 | | ACUGUAGUAUGGGCACUUCCAG |
| hsa-miR-325 | 16 | 241 | A16 | | CCUAGUAGGUGUCCAGUAAGUGU |
| hsa-miR-509-3-5p | 17 | 257 | A17 | | UACUGCAGACGUGGCAAUCAUG |
| hsa-miR-210-3p | 18 | 273 | A18 | | CUGUGCGUGUGACAGCGGCUGA |
| hsa-miR-199b-5p | 19 | 289 | A19 | | CCCAGUGUUUAGACUAUCUGUUC |
| hsa-miR-194-5p | 20 | 305 | A20 | | UGUAACAGCAACUCCAUGUGGA |
| hsa-let-7g-5p | 21 | 321 | A21 | | UGAGGUAGUAGUUUGUACAGUU |
| hsa-miR-203a | 22 | 337 | A22 | | GUGAAAUGUUUAGGACCACUAG |
| hsa-miR-181a-3p | 23 | 353 | A23 | | ACCAUCGACCGUUGAUUGUACC |
| hsa-miR-137 | 24 | 369 | A24 | | UUAUUGCUUAAGAAUACGCGUAG |
| hsa-miR-551b-3p | 25 | 2 | B01 | | GCGACCCAUACUUGGUUUCAG |
| Blank (H2O) | 26 | 18 | B02 | |  |
| hsa-miR-524-3p | 27 | 34 | B03 | | GAAGGCGCUUCCCUUUGGAGU |
| UniSp2 | 28 | 50 | B04 | |  |
| hsa-miR-486-5p | 29 | 66 | B05 | | UCCUGUACUGAGCUGCCCCGAG |
| UniSp4 | 30 | 82 | B06 | |  |
| UniSp3 IPC | 31 | 98 | B07 | |  |
| UniSp5 | 32 | 114 | B08 | |  |
| hsa-miR-329-3p | 33 | 130 | B09 | | AACACACCUGGUUAACCUCUUU |
| UniSp6 | 34 | 146 | B10 | |  |
| hsa-miR-487b-3p | 35 | 162 | B11 | | AAUCGUACAGGGUCAUCCACUU |
| cel-miR-39-3p | 36 | 178 | B12 | |  |
| hsa-miR-138-5p | 37 | 194 | B13 | | AGCUGGUGUUGUGAAUCAGGCCG |
| hsa-miR-191-5p | 38 | 210 | B14 | | CAACGGAAUCCCAAAAGCAGCUG |
| mmu-miR-378a-3p | 39 | 226 | B15 | | ACUGGACUUGGAGUCAGAAGG |
| hsa-miR-103a-3p | 40 | 242 | B16 | | AGCAGCAUUGUACAGGGCUAUGA |
| hsa-miR-890 | 41 | 258 | B17 | | UACUUGGAAAGGCAUCAGUUG |
| hsa-miR-423-5p | 42 | 274 | B18 | | UGAGGGGCAGAGAGCGAGACUUU |
| hsa-miR-221-3p | 43 | 290 | B19 | | AGCUACAUUGUCUGCUGGGUUUC |
| SNORD38B | 44 | 306 | B20 | |  |
| hsa-miR-301b | 45 | 322 | B21 | | CAGUGCAAUGAUAUUGUCAAAGC |
| SNORD49A | 46 | 338 | B22 | |  |
| hsa-miR-550a-5p | 47 | 354 | B23 | | AGUGCCUGAGGGAGUAAGAGCCC |
| U6 snRNA | 48 | 370 | B24 | |  |
| hsa-miR-532-5p | 49 | 3 | C01 | | CAUGCCUUGAGUGUAGGACCGU |
| hsa-miR-99a-5p | 50 | 19 | C02 | | AACCCGUAGAUCCGAUCUUGUG |
| hsa-miR-16-5p | 51 | 35 | C03 | | UAGCAGCACGUAAAUAUUGGCG |
| hsa-miR-98-5p | 52 | 51 | C04 | | UGAGGUAGUAAGUUGUAUUGUU |
| hsa-miR-185-5p | 53 | 67 | C05 | | UGGAGAGAAAGGCAGUUCCUGA |
| hsa-miR-25-3p | 54 | 83 | C06 | | CAUUGCACUUGUCUCGGUCUGA |
| UniSp3 IPC | 55 | 99 | C07 | |  |
| UniSp3 IPC | 56 | 115 | C08 | |  |
| hsa-miR-765 | 57 | 131 | C09 | | UGGAGGAGAAGGAAGGUGAUG |
| hsa-miR-24-3p | 58 | 147 | C10 | | UGGCUCAGUUCAGCAGGAACAG |
| hsa-miR-369-5p | 59 | 163 | C11 | | AGAUCGACCGUGUUAUAUUCGC |
| hsa-miR-425-5p | 60 | 179 | C12 | | AAUGACACGAUCACUCCCGUUGA |
| hsa-miR-590-5p | 61 | 195 | C13 | | GAGCUUAUUCAUAAAAGUGCAG |
| hsa-miR-760 | 62 | 211 | C14 | | CGGCUCUGGGUCUGUGGGGA |
| hsa-miR-574-3p | 63 | 227 | C15 | | CACGCUCAUGCACACACCCACA |
| hsa-miR-130b-3p | 64 | 243 | C16 | | CAGUGCAAUGAUGAAAGGGCAU |
| hsa-miR-30c-5p | 65 | 259 | C17 | | UGUAAACAUCCUACACUCUCAGC |
| hsa-miR-133b | 66 | 275 | C18 | | UUUGGUCCCCUUCAACCAGCUA |
| hsa-miR-524-5p | 67 | 291 | C19 | | CUACAAAGGGAAGCACUUUCUC |
| hsa-miR-23a-3p | 68 | 307 | C20 | | AUCACAUUGCCAGGGAUUUCC |
| hsa-miR-193b-3p | 69 | 323 | C21 | | AACUGGCCCUCAAAGUCCCGCU |
| hsa-miR-501-5p | 70 | 339 | C22 | | AAUCCUUUGUCCCUGGGUGAGA |
| hsa-miR-518c-5p | 71 | 355 | C23 | | UCUCUGGAGGGAAGCACUUUCUG |
| hsa-miR-130a-3p | 72 | 371 | C24 | | CAGUGCAAUGUUAAAAGGGCAU |
| hsa-miR-933 | 73 | 4 | D01 | | UGUGCGCAGGGAGACCUCUCCC |
| hsa-miR-379-5p | 74 | 20 | D02 | | UGGUAGACUAUGGAACGUAGG |
| hsa-miR-452-5p | 75 | 36 | D03 | | AACUGUUUGCAGAGGAAACUGA |
| hsa-miR-589-5p | 76 | 52 | D04 | | UGAGAACCACGUCUGCUCUGAG |
| hsa-miR-141-3p | 77 | 68 | D05 | | UAACACUGUCUGGUAAAGAUGG |
| hsa-miR-342-3p | 78 | 84 | D06 | | UCUCACACAGAAAUCGCACCCGU |
| hsa-miR-668-3p | 79 | 100 | D07 | | UGUCACUCGGCUCGGCCCACUAC |
| hsa-miR-934 | 80 | 116 | D08 | | UGUCUACUACUGGAGACACUGG |
| hsa-miR-101-3p | 81 | 132 | D09 | | UACAGUACUGUGAUAACUGAA |
| hsa-miR-539-5p | 82 | 148 | D10 | | GGAGAAAUUAUCCUUGGUGUGU |
| hsa-miR-331-3p | 83 | 164 | D11 | | GCCCCUGGGCCUAUCCUAGAA |
| hsa-miR-499a-5p | 84 | 180 | D12 | | UUAAGACUUGCAGUGAUGUUU |
| hsa-miR-196a-5p | 85 | 196 | D13 | | UAGGUAGUUUCAUGUUGUUGGG |
| hsa-miR-888-5p | 86 | 212 | D14 | | UACUCAAAAAGCUGUCAGUCA |
| hsa-miR-330-3p | 87 | 228 | D15 | | GCAAAGCACACGGCCUGCAGAGA |
| hsa-miR-570-3p | 88 | 244 | D16 | | CGAAAACAGCAAUUACCUUUGC |
| hsa-miR-518c-3p | 89 | 260 | D17 | | CAAAGCGCUUCUCUUUAGAGUGU |
| hsa-miR-200a-3p | 90 | 276 | D18 | | UAACACUGUCUGGUAACGAUGU |
| hsa-miR-188-5p | 91 | 292 | D19 | | CAUCCCUUGCAUGGUGGAGGG |
| hsa-miR-26a-5p | 92 | 308 | D20 | | UUCAAGUAAUCCAGGAUAGGCU |
| hsa-miR-99b-5p | 93 | 324 | D21 | | CACCCGUAGAACCGACCUUGCG |
| hsa-miR-431-5p | 94 | 340 | D22 | | UGUCUUGCAGGCCGUCAUGCA |
| hsa-miR-23b-3p | 95 | 356 | D23 | | AUCACAUUGCCAGGGAUUACC |
| hsa-miR-367-3p | 96 | 372 | D24 | | AAUUGCACUUUAGCAAUGGUGA |
| hsa-miR-505-3p | 97 | 5 | E01 | | CGUCAACACUUGCUGGUUUCCU |
| hsa-miR-18a-5p | 98 | 21 | E02 | | UAAGGUGCAUCUAGUGCAGAUAG |
| hsa-miR-92a-3p | 99 | 37 | E03 | | UAUUGCACUUGUCCCGGCCUGU |
| hsa-miR-500a-5p | 100 | 53 | E04 | | UAAUCCUUGCUACCUGGGUGAGA |
| hsa-miR-887-3p | 101 | 69 | E05 | | GUGAACGGGCGCCAUCCCGAGG |
| hsa-miR-491-3p | 102 | 85 | E06 | | CUUAUGCAAGAUUCCCUUCUAC |
| hsa-miR-423-3p | 103 | 101 | E07 | | AGCUCGGUCUGAGGCCCCUCAGU |
| hsa-miR-126-3p | 104 | 117 | E08 | | UCGUACCGUGAGUAAUAAUGCG |
| hsa-miR-421 | 105 | 133 | E09 | | AUCAACAGACAUUAAUUGGGCGC |
| hsa-miR-376b-3p | 106 | 149 | E10 | | AUCAUAGAGGAAAAUCCAUGUU |
| hsa-miR-302c-3p | 107 | 165 | E11 | | UAAGUGCUUCCAUGUUUCAGUGG |
| hsa-miR-625-3p | 108 | 181 | E12 | | GACUAUAGAACUUUCCCCCUCA |
| hsa-miR-339-5p | 109 | 197 | E13 | | UCCCUGUCCUCCAGGAGCUCACG |
| hsa-miR-873-5p | 110 | 213 | E14 | | GCAGGAACUUGUGAGUCUCCU |
| hsa-miR-323a-3p | 111 | 229 | E15 | | CACAUUACACGGUCGACCUCU |
| hsa-miR-181d-5p | 112 | 245 | E16 | | AACAUUCAUUGUUGUCGGUGGGU |
| hsa-miR-125a-5p | 113 | 261 | E17 | | UCCCUGAGACCCUUUAACCUGUGA |
| hsa-miR-129-5p | 114 | 277 | E18 | | CUUUUUGCGGUCUGGGCUUGC |
| hsa-miR-492 | 115 | 293 | E19 | | AGGACCUGCGGGACAAGAUUCUU |
| hsa-miR-20a-5p | 116 | 309 | E20 | | UAAAGUGCUUAUAGUGCAGGUAG |
| hsa-miR-374b-5p | 117 | 325 | E21 | | AUAUAAUACAACCUGCUAAGUG |
| hsa-miR-302d-3p | 118 | 341 | E22 | | UAAGUGCUUCCAUGUUUGAGUGU |
| hsa-miR-346 | 119 | 357 | E23 | | UGUCUGCCCGCAUGCCUGCCUCU |
| hsa-miR-151a-3p | 120 | 373 | E24 | | CUAGACUGAAGCUCCUUGAGG |
| hsa-miR-493-3p | 121 | 6 | F01 | | UGAAGGUCUACUGUGUGCCAGG |
| hsa-miR-122-5p | 122 | 22 | F02 | | UGGAGUGUGACAAUGGUGUUUG |
| hsa-miR-99a-3p | 123 | 38 | F03 | | CAAGCUCGCUUCUAUGGGUCUG |
| hsa-miR-361-5p | 124 | 54 | F04 | | UUAUCAGAAUCUCCAGGGGUAC |
| hsa-miR-202-3p | 125 | 70 | F05 | | AGAGGUAUAGGGCAUGGGAA |
| hsa-miR-125b-5p | 126 | 86 | F06 | | UCCCUGAGACCCUAACUUGUGA |
| hsa-miR-503-5p | 127 | 102 | F07 | | UAGCAGCGGGAACAGUUCUGCAG |
| hsa-miR-204-5p | 128 | 118 | F08 | | UUCCCUUUGUCAUCCUAUGCCU |
| hsa-miR-30d-5p | 129 | 134 | F09 | | UGUAAACAUCCCCGACUGGAAG |
| hsa-miR-301a-3p | 130 | 150 | F10 | | CAGUGCAAUAGUAUUGUCAAAGC |
| hsa-miR-362-5p | 131 | 166 | F11 | | AAUCCUUGGAACCUAGGUGUGAGU |
| hsa-miR-30b-3p | 132 | 182 | F12 | | CUGGGAGGUGGAUGUUUACUUC |
| hsa-miR-654-5p | 133 | 198 | F13 | | UGGUGGGCCGCAGAACAUGUGC |
| hsa-miR-545-3p | 134 | 214 | F14 | | UCAGCAAACAUUUAUUGUGUGC |
| hsa-miR-29b-2-5p | 135 | 230 | F15 | | CUGGUUUCACAUGGUGGCUUAG |
| hsa-miR-491-5p | 136 | 246 | F16 | | AGUGGGGAACCCUUCCAUGAGG |
| hsa-miR-92b-3p | 137 | 262 | F17 | | UAUUGCACUCGUCCCGGCCUCC |
| hsa-miR-665 | 138 | 278 | F18 | | ACCAGGAGGCUGAGGCCCCU |
| hsa-miR-506-3p | 139 | 294 | F19 | | UAAGGCACCCUUCUGAGUAGA |
| hsa-miR-363-3p | 140 | 310 | F20 | | AAUUGCACGGUAUCCAUCUGUA |
| hsa-miR-132-3p | 141 | 326 | F21 | | UAACAGUCUACAGCCAUGGUCG |
| hsa-miR-651-5p | 142 | 342 | F22 | | UUUAGGAUAAGCUUGACUUUUG |
| hsa-miR-628-3p | 143 | 358 | F23 | | UCUAGUAAGAGUGGCAGUCGA |
| hsa-miR-432-5p | 144 | 374 | F24 | | UCUUGGAGUAGGUCAUUGGGUGG |
| hsa-miR-154-3p | 145 | 7 | G01 | | AAUCAUACACGGUUGACCUAUU |
| hsa-miR-27a-3p | 146 | 23 | G02 | | UUCACAGUGGCUAAGUUCCGC |
| hsa-miR-376c-3p | 147 | 39 | G03 | | AACAUAGAGGAAAUUCCACGU |
| hsa-miR-940 | 148 | 55 | G04 | | AAGGCAGGGCCCCCGCUCCCC |
| hsa-miR-22-5p | 149 | 71 | G05 | | AGUUCUUCAGUGGCAAGCUUUA |
| hsa-miR-224-5p | 150 | 87 | G06 | | CAAGUCACUAGUGGUUCCGUU |
| hsa-miR-885-5p | 151 | 103 | G07 | | UCCAUUACACUACCCUGCCUCU |
| hsa-miR-320a | 152 | 119 | G08 | | AAAAGCUGGGUUGAGAGGGCGA |
| hsa-miR-18b-5p | 153 | 135 | G09 | | UAAGGUGCAUCUAGUGCAGUUAG |
| hsa-miR-187-3p | 154 | 151 | G10 | | UCGUGUCUUGUGUUGCAGCCGG |
| hsa-miR-516b-5p | 155 | 167 | G11 | | AUCUGGAGGUAAGAAGCACUUU |
| hsa-miR-302c-5p | 156 | 183 | G12 | | UUUAACAUGGGGGUACCUGCUG |
| hsa-miR-548b-3p | 157 | 199 | G13 | | CAAGAACCUCAGUUGCUUUUGU |
| hsa-miR-186-5p | 158 | 215 | G14 | | CAAAGAAUUCUCCUUUUGGGCU |
| hsa-miR-199a-5p | 159 | 231 | G15 | | CCCAGUGUUCAGACUACCUGUUC |
| hsa-miR-155-5p | 160 | 247 | G16 | | UUAAUGCUAAUCGUGAUAGGGGU |
| hsa-miR-107 | 161 | 263 | G17 | | AGCAGCAUUGUACAGGGCUAUCA |
| hsa-miR-302b-3p | 162 | 279 | G18 | | UAAGUGCUUCCAUGUUUUAGUAG |
| hsa-miR-662 | 163 | 295 | G19 | | UCCCACGUUGUGGCCCAGCAG |
| hsa-miR-519d-3p | 164 | 311 | G20 | | CAAAGUGCCUCCCUUUAGAGUG |
| hsa-miR-485-3p | 165 | 327 | G21 | | GUCAUACACGGCUCUCCUCUCU |
| hsa-miR-200b-3p | 166 | 343 | G22 | | UAAUACUGCCUGGUAAUGAUGA |
| hsa-miR-337-3p | 167 | 359 | G23 | | CUCCUAUAUGAUGCCUUUCUUC |
| hsa-miR-494-3p | 168 | 375 | G24 | | UGAAACAUACACGGGAAACCUC |
| hsa-miR-371a-3p | 169 | 8 | H01 | | AAGUGCCGCCAUCUUUUGAGUGU |
| hsa-miR-637 | 170 | 24 | H02 | | ACUGGGGGCUUUCGGGCUCUGCGU |
| hsa-miR-144-3p | 171 | 40 | H03 | | UACAGUAUAGAUGAUGUACU |
| hsa-miR-16-1-3p | 172 | 56 | H04 | | CCAGUAUUAACUGUGCUGCUGA |
| hsa-miR-631 | 173 | 72 | H05 | | AGACCUGGCCCAGACCUCAGC |
| hsa-miR-34c-5p | 174 | 88 | H06 | | AGGCAGUGUAGUUAGCUGAUUGC |
| hsa-miR-211-5p | 175 | 104 | H07 | | UUCCCUUUGUCAUCCUUCGCCU |
| hsa-miR-454-3p | 176 | 120 | H08 | | UAGUGCAAUAUUGCUUAUAGGGU |
| hsa-let-7f-5p | 177 | 136 | H09 | | UGAGGUAGUAGAUUGUAUAGUU |
| hsa-miR-30e-5p | 178 | 152 | H10 | | UGUAAACAUCCUUGACUGGAAG |
| hsa-miR-34a-5p | 179 | 168 | H11 | | UGGCAGUGUCUUAGCUGGUUGU |
| hsa-miR-663a | 180 | 184 | H12 | | AGGCGGGGCGCCGCGGGACCGC |
| hsa-miR-518e-3p | 181 | 200 | H13 | | AAAGCGCUUCCCUUCAGAGUG |
| hsa-miR-29b-3p | 182 | 216 | H14 | | UAGCACCAUUUGAAAUCAGUGUU |
| hsa-miR-658 | 183 | 232 | H15 | | GGCGGAGGGAAGUAGGUCCGUUGGU |
| hsa-miR-572 | 184 | 248 | H16 | | GUCCGCUCGGCGGUGGCCCA |
| hsa-miR-802 | 185 | 264 | H17 | | CAGUAACAAAGAUUCAUCCUUGU |
| hsa-miR-521 | 186 | 280 | H18 | | AACGCACUUCCCUUUAGAGUGU |
| hsa-miR-433-3p | 187 | 296 | H19 | | AUCAUGAUGGGCUCCUCGGUGU |
| hsa-miR-660-5p | 188 | 312 | H20 | | UACCCAUUGCAUAUCGGAGUUG |
| hsa-let-7c-5p | 189 | 328 | H21 | | UGAGGUAGUAGGUUGUAUGGUU |
| hsa-miR-28-5p | 190 | 344 | H22 | | AAGGAGCUCACAGUCUAUUGAG |
| hsa-miR-324-5p | 191 | 360 | H23 | | CGCAUCCCCUAGGGCAUUGGUGU |
| hsa-miR-219a-5p | 192 | 376 | H24 | | UGAUUGUCCAAACGCAAUUCU |
| hsa-miR-19b-3p | 193 | 9 | I01 | | UGUGCAAAUCCAUGCAAAACUGA |
| hsa-miR-526b-5p | 194 | 25 | I02 | | CUCUUGAGGGAAGCACUUUCUGU |
| hsa-miR-215-5p | 195 | 41 | I03 | | AUGACCUAUGAAUUGACAGAC |
| hsa-miR-30b-5p | 196 | 57 | I04 | | UGUAAACAUCCUACACUCAGCU |
| hsa-miR-184 | 197 | 73 | I05 | | UGGACGGAGAACUGAUAAGGGU |
| hsa-miR-422a | 198 | 89 | I06 | | ACUGGACUUAGGGUCAGAAGGC |
| hsa-miR-199a-3p | 199 | 105 | I07 | | ACAGUAGUCUGCACAUUGGUUA |
| hsa-miR-335-5p | 200 | 121 | I08 | | UCAAGAGCAAUAACGAAAAAUGU |
| hsa-miR-519a-3p | 201 | 137 | I09 | | AAAGUGCAUCCUUUUAGAGUGU |
| hsa-miR-21-5p | 202 | 153 | I10 | | UAGCUUAUCAGACUGAUGUUGA |
| hsa-miR-129-2-3p | 203 | 169 | I11 | | AAGCCCUUACCCCAAAAAGCAU |
| hsa-miR-26b-5p | 204 | 185 | I12 | | UUCAAGUAAUUCAGGAUAGGU |
| hsa-miR-214-3p | 205 | 201 | I13 | | ACAGCAGGCACAGACAGGCAGU |
| hsa-miR-32-5p | 206 | 217 | I14 | | UAUUGCACAUUACUAAGUUGCA |
| hsa-miR-324-3p | 207 | 233 | I15 | | ACUGCCCCAGGUGCUGCUGG |
| hsa-miR-488-3p | 208 | 249 | I16 | | UUGAAAGGCUAUUUCUUGGUC |
| hsa-miR-371a-5p | 209 | 265 | I17 | | ACUCAAACUGUGGGGGCACU |
| hsa-miR-455-5p | 210 | 281 | I18 | | UAUGUGCCUUUGGACUACAUCG |
| hsa-miR-891a-5p | 211 | 297 | I19 | | UGCAACGAACCUGAGCCACUGA |
| hsa-miR-549a | 212 | 313 | I20 | | UGACAACUAUGGAUGAGCUCU |
| hsa-miR-205-5p | 213 | 329 | I21 | | UCCUUCAUUCCACCGGAGUCUG |
| hsa-miR-518b | 214 | 345 | I22 | | CAAAGCGCUCCCCUUUAGAGGU |
| hsa-miR-19a-3p | 215 | 361 | I23 | | UGUGCAAAUCUAUGCAAAACUGA |
| hsa-miR-150-5p | 216 | 377 | I24 | | UCUCCCAACCCUUGUACCAGUG |
| hsa-miR-15a-5p | 217 | 10 | J01 | | UAGCAGCACAUAAUGGUUUGUG |
| hsa-let-7d-3p | 218 | 26 | J02 | | CUAUACGACCUGCUGCCUUUCU |
| hsa-miR-608 | 219 | 42 | J03 | | AGGGGUGGUGUUGGGACAGCUCCGU |
| hsa-miR-671-5p | 220 | 58 | J04 | | AGGAAGCCCUGGAGGGGCUGGAG |
| hsa-miR-497-5p | 221 | 74 | J05 | | CAGCAGCACACUGUGGUUUGU |
| hsa-miR-877-5p | 222 | 90 | J06 | | GUAGAGGAGAUGGCGCAGGG |
| hsa-miR-187-5p | 223 | 106 | J07 | | GGCUACAACACAGGACCCGGGC |
| hsa-miR-10b-5p | 224 | 122 | J08 | | UACCCUGUAGAACCGAAUUUGUG |
| hsa-let-7i-5p | 225 | 138 | J09 | | UGAGGUAGUAGUUUGUGCUGUU |
| hsa-miR-202-5p | 226 | 154 | J10 | | UUCCUAUGCAUAUACUUCUUUG |
| hsa-miR-652-3p | 227 | 170 | J11 | | AAUGGCGCCACUAGGGUUGUG |
| hsa-miR-126-5p | 228 | 186 | J12 | | CAUUAUUACUUUUGGUACGCG |
| hsa-miR-30e-3p | 229 | 202 | J13 | | CUUUCAGUCGGAUGUUUACAGC |
| hsa-miR-181c-5p | 230 | 218 | J14 | | AACAUUCAACCUGUCGGUGAGU |
| hsa-miR-9-3p | 231 | 234 | J15 | | AUAAAGCUAGAUAACCGAAAGU |
| hsa-miR-548c-3p | 232 | 250 | J16 | | CAAAAAUCUCAAUUACUUUUGC |
| hsa-miR-152-3p | 233 | 266 | J17 | | UCAGUGCAUGACAGAACUUGG |
| hsa-miR-93-5p | 234 | 282 | J18 | | CAAAGUGCUGUUCGUGCAGGUAG |
| hsa-miR-365a-3p | 235 | 298 | J19 | | UAAUGCCCCUAAAAAUCCUUAU |
| hsa-miR-29c-3p | 236 | 314 | J20 | | UAGCACCAUUUGAAAUCGGUUA |
| hsa-miR-372-3p | 237 | 330 | J21 | | AAAGUGCUGCGACAUUUGAGCGU |
| hsa-miR-133a-3p | 238 | 346 | J22 | | UUUGGUCCCCUUCAACCAGCUG |
| hsa-miR-124-3p | 239 | 362 | J23 | | UAAGGCACGCGGUGAAUGCC |
| hsa-miR-190a-5p | 240 | 378 | J24 | | UGAUAUGUUUGAUAUAUUAGGU |
| hsa-miR-302a-3p | 241 | 11 | K01 | | UAAGUGCUUCCAUGUUUUGGUGA |
| hsa-miR-595 | 242 | 27 | K02 | | GAAGUGUGCCGUGGUGUGUCU |
| hsa-miR-602 | 243 | 43 | K03 | | GACACGGGCGACAGCUGCGGCCC |
| hsa-miR-223-3p | 244 | 59 | K04 | | UGUCAGUUUGUCAAAUACCCCA |
| hsa-miR-627-5p | 245 | 75 | K05 | | GUGAGUCUCUAAGAAAAGAGGA |
| hsa-miR-34b-3p | 246 | 91 | K06 | | CAAUCACUAACUCCACUGCCAU |
| hsa-miR-410-3p | 247 | 107 | K07 | | AAUAUAACACAGAUGGCCUGU |
| hsa-miR-17-5p | 248 | 123 | K08 | | CAAAGUGCUUACAGUGCAGGUAG |
| hsa-miR-376a-3p | 249 | 139 | K09 | | AUCAUAGAGGAAAAUCCACGU |
| hsa-miR-514a-3p | 250 | 155 | K10 | | AUUGACACUUCUGUGAGUAGA |
| hsa-miR-512-5p | 251 | 171 | K11 | | CACUCAGCCUUGAGGGCACUUUC |
| hsa-miR-449a | 252 | 187 | K12 | | UGGCAGUGUAUUGUUAGCUGGU |
| hsa-miR-498 | 253 | 203 | K13 | | UUUCAAGCCAGGGGGCGUUUUUC |
| hsa-miR-148b-3p | 254 | 219 | K14 | | UCAGUGCAUCACAGAACUUUGU |
| hsa-miR-127-3p | 255 | 235 | K15 | | UCGGAUCCGUCUGAGCUUGGCU |
| hsa-miR-598-3p | 256 | 251 | K16 | | UACGUCAUCGUUGUCAUCGUCA |
| hsa-miR-96-5p | 257 | 267 | K17 | | UUUGGCACUAGCACAUUUUUGCU |
| hsa-let-7d-5p | 258 | 283 | K18 | | AGAGGUAGUAGGUUGCAUAGUU |
| hsa-miR-135b-5p | 259 | 299 | K19 | | UAUGGCUUUUCAUUCCUAUGUGA |
| hsa-miR-495-3p | 260 | 315 | K20 | | AAACAAACAUGGUGCACUUCUU |
| hsa-miR-299-5p | 261 | 331 | K21 | | UGGUUUACCGUCCCACAUACAU |
| hsa-miR-34c-3p | 262 | 347 | K22 | | AAUCACUAACCACACGGCCAGG |
| hsa-miR-596 | 263 | 363 | K23 | | AAGCCUGCCCGGCUCCUCGGG |
| hsa-miR-744-5p | 264 | 379 | K24 | | UGCGGGGCUAGGGCUAACAGCA |
| hsa-miR-145-5p | 265 | 12 | L01 | | GUCCAGUUUUCCCAGGAAUCCCU |
| hsa-miR-622 | 266 | 28 | L02 | | ACAGUCUGCUGAGGUUGGAGC |
| hsa-miR-516a-5p | 267 | 44 | L03 | | UUCUCGAGGAAAGAAGCACUUUC |
| hsa-let-7a-5p | 268 | 60 | L04 | | UGAGGUAGUAGGUUGUAUAGUU |
| hsa-miR-96-3p | 269 | 76 | L05 | | AAUCAUGUGCAGUGCCAAUAUG |
| hsa-miR-185-3p | 270 | 92 | L06 | | AGGGGCUGGCUUUCCUCUGGUC |
| hsa-miR-615-3p | 271 | 108 | L07 | | UCCGAGCCUGGGUCUCCCUCUU |
| hsa-miR-128-3p | 272 | 124 | L08 | | UCACAGUGAACCGGUCUCUUU |
| hsa-miR-766-3p | 273 | 140 | L09 | | ACUCCAGCCCCACAGCCUCAGC |
| hsa-miR-206 | 274 | 156 | L10 | | UGGAAUGUAAGGAAGUGUGUGG |
| hsa-miR-298 | 275 | 172 | L11 | | AGCAGAAGCAGGGAGGUUCUCCCA |
| hsa-miR-193a-5p | 276 | 188 | L12 | | UGGGUCUUUGCGGGCGAGAUGA |
| hsa-miR-449b-5p | 277 | 204 | L13 | | AGGCAGUGUAUUGUUAGCUGGC |
| hsa-miR-520d-5p | 278 | 220 | L14 | | CUACAAAGGGAAGCCCUUUC |
| hsa-miR-192-5p | 279 | 236 | L15 | | CUGACCUAUGAAUUGACAGCC |
| hsa-miR-29a-3p | 280 | 252 | L16 | | UAGCACCAUCUGAAAUCGGUUA |
| hsa-miR-18a-3p | 281 | 268 | L17 | | ACUGCCCUAAGUGCUCCUUCUGG |
| hsa-miR-383-5p | 282 | 284 | L18 | | AGAUCAGAAGGUGAUUGUGGCU |
| hsa-miR-9-5p | 283 | 300 | L19 | | UCUUUGGUUAUCUAGCUGUAUGA |
| hsa-miR-142-5p | 284 | 316 | L20 | | CAUAAAGUAGAAAGCACUACU |
| hsa-miR-363-5p | 285 | 332 | L21 | | CGGGUGGAUCACGAUGCAAUUU |
| hsa-miR-147b | 286 | 348 | L22 | | GUGUGCGGAAAUGCUUCUGCUA |
| hsa-miR-197-3p | 287 | 364 | L23 | | UUCACCACCUUCUCCACCCAGC |
| hsa-miR-597-5p | 288 | 380 | L24 | | UGUGUCACUCGAUGACCACUGU |
| hsa-miR-326 | 289 | 13 | M01 | | CCUCUGGGCCCUUCCUCCAG |
| hsa-miR-15b-5p | 290 | 29 | M02 | | UAGCAGCACAUCAUGGUUUACA |
| hsa-miR-105-5p | 291 | 45 | M03 | | UCAAAUGCUCAGACUCCUGUGGU |
| hsa-miR-196b-5p | 292 | 61 | M04 | | UAGGUAGUUUCCUGUUGUUGGG |
| hsa-miR-296-5p | 293 | 77 | M05 | | AGGGCCCCCCCUCAAUCCUGU |
| hsa-miR-20b-5p | 294 | 93 | M06 | | CAAAGUGCUCAUAGUGCAGGUAG |
| hsa-miR-147a | 295 | 109 | M07 | | GUGUGUGGAAAUGCUUCUGC |
| hsa-miR-198 | 296 | 125 | M08 | | GGUCCAGAGGGGAGAUAGGUUC |
| hsa-miR-375 | 297 | 141 | M09 | | UUUGUUCGUUCGGCUCGCGUGA |
| hsa-miR-517a-3p | 298 | 157 | M10 | | AUCGUGCAUCCCUUUAGAGUGU |
| hsa-miR-361-3p | 299 | 173 | M11 | | UCCCCCAGGUGUGAUUCUGAUUU |
| hsa-miR-21-3p | 300 | 189 | M12 | | CAACACCAGUCGAUGGGCUGU |
| hsa-miR-373-3p | 301 | 205 | M13 | | GAAGUGCUUCGAUUUUGGGGUGU |
| hsa-miR-518f-3p | 302 | 221 | M14 | | GAAAGCGCUUCUCUUUAGAGG |
| hsa-miR-222-3p | 303 | 237 | M15 | | AGCUACAUCUGGCUACUGGGU |
| hsa-miR-617 | 304 | 253 | M16 | | AGACUUCCCAUUUGAAGGUGGC |
| hsa-miR-154-5p | 305 | 269 | M17 | | UAGGUUAUCCGUGUUGCCUUCG |
| hsa-miR-708-5p | 306 | 285 | M18 | | AAGGAGCUUACAAUCUAGCUGGG |
| hsa-let-7b-5p | 307 | 301 | M19 | | UGAGGUAGUAGGUUGUGUGGUU |
| hsa-miR-95-3p | 308 | 317 | M20 | | UUCAACGGGUAUUUAUUGAGCA |
| hsa-miR-517c-3p | 309 | 333 | M21 | | AUCGUGCAUCCUUUUAGAGUGU |
| hsa-miR-151a-5p | 310 | 349 | M22 | | UCGAGGAGCUCACAGUCUAGU |
| hsa-miR-502-5p | 311 | 365 | M23 | | AUCCUUGCUAUCUGGGUGCUA |
| hsa-miR-345-5p | 312 | 381 | M24 | | GCUGACUCCUAGUCCAGGGCUC |
| hsa-miR-509-3p | 313 | 14 | N01 | | UGAUUGGUACGUCUGUGGGUAG |
| hsa-miR-134-5p | 314 | 30 | N02 | | UGUGACUGGUUGACCAGAGGGG |
| hsa-miR-382-5p | 315 | 46 | N03 | | GAAGUUGUUCGUGGUGGAUUCG |
| hsa-miR-490-3p | 316 | 62 | N04 | | CAACCUGGAGGACUCCAUGCUG |
| hsa-miR-200c-3p | 317 | 78 | N05 | | UAAUACUGCCGGGUAAUGAUGGA |
| hsa-miR-30a-5p | 318 | 94 | N06 | | UGUAAACAUCCUCGACUGGAAG |
| hsa-miR-181b-5p | 319 | 110 | N07 | | AACAUUCAUUGCUGUCGGUGGGU |
| hsa-miR-33a-5p | 320 | 126 | N08 | | GUGCAUUGUAGUUGCAUUGCA |
| hsa-miR-195-5p | 321 | 142 | N09 | | UAGCAGCACAGAAAUAUUGGC |
| hsa-miR-874-3p | 322 | 158 | N10 | | CUGCCCUGGCCCGAGGGACCGA |
| hsa-miR-135a-5p | 323 | 174 | N11 | | UAUGGCUUUUUAUUCCUAUGUGA |
| hsa-miR-26a-2-3p | 324 | 190 | N12 | | CCUAUUCUUGAUUACUUGUUUC |
| hsa-miR-146b-5p | 325 | 206 | N13 | | UGAGAACUGAAUUCCAUAGGCU |
| hsa-miR-412-3p | 326 | 222 | N14 | | ACUUCACCUGGUCCACUAGCCGU |
| hsa-miR-1 | 327 | 238 | N15 | | UGGAAUGUAAAGAAGUAUGUAU |
| hsa-miR-299-3p | 328 | 254 | N16 | | UAUGUGGGAUGGUAAACCGCUU |
| hsa-miR-142-3p | 329 | 270 | N17 | | UGUAGUGUUUCCUACUUUAUGGA |
| hsa-miR-338-3p | 330 | 286 | N18 | | UCCAGCAUCAGUGAUUUUGUUG |
| hsa-miR-584-5p | 331 | 302 | N19 | | UUAUGGUUUGCCUGGGACUGAG |
| hsa-miR-377-3p | 332 | 318 | N20 | | AUCACACAAAGGCAACUUUUGU |
| hsa-miR-216a-5p | 333 | 334 | N21 | | UAAUCUCAGCUGGCAACUGUGA |
| hsa-miR-424-5p | 334 | 350 | N22 | | CAGCAGCAAUUCAUGUUUUGAA |
| hsa-miR-921 | 335 | 366 | N23 | | CUAGUGAGGGACAGAACCAGGAUUC |
| hsa-miR-513a-5p | 336 | 382 | N24 | | UUCACAGGGAGGUGUCAU |
| hsa-miR-140-3p | 337 | 15 | O01 | | UACCACAGGGUAGAACCACGG |
| hsa-miR-181a-5p | 338 | 31 | O02 | | AACAUUCAACGCUGUCGGUGAGU |
| hsa-miR-10a-5p | 339 | 47 | O03 | | UACCCUGUAGAUCCGAAUUUGUG |
| hsa-miR-106a-5p | 340 | 63 | O04 | | AAAAGUGCUUACAGUGCAGGUAG |
| hsa-miR-182-5p | 341 | 79 | O05 | | UUUGGCAAUGGUAGAACUCACACU |
| hsa-miR-370-3p | 342 | 95 | O06 | | GCCUGCUGGGGUGGAACCUGGU |
| hsa-miR-576-5p | 343 | 111 | O07 | | AUUCUAAUUUCUCCACGUCUUU |
| hsa-miR-425-3p | 344 | 127 | O08 | | AUCGGGAAUGUCGUGUCCGCCC |
| hsa-miR-450a-5p | 345 | 143 | O09 | | UUUUGCGAUGUGUUCCUAAUAU |
| hsa-miR-411-5p | 346 | 159 | O10 | | UAGUAGACCGUAUAGCGUACG |
| hsa-miR-216b-5p | 347 | 175 | O11 | | AAAUCUCUGCAGGCAAAUGUGA |
| hsa-miR-106b-5p | 348 | 191 | O12 | | UAAAGUGCUGACAGUGCAGAU |
| hsa-miR-22-3p | 349 | 207 | O13 | | AAGCUGCCAGUUGAAGAACUGU |
| hsa-miR-510-5p | 350 | 223 | O14 | | UACUCAGGAGAGUGGCAAUCAC |
| hsa-miR-212-3p | 351 | 239 | O15 | | UAACAGUCUCCAGUCACGGCC |
| hsa-miR-525-5p | 352 | 255 | O16 | | CUCCAGAGGGAUGCACUUUCU |
| hsa-miR-542-5p | 353 | 271 | O17 | | UCGGGGAUCAUCAUGUCACGAGA |
| hsa-miR-576-3p | 354 | 287 | O18 | | AAGAUGUGGAAAAAUUGGAAUC |
| hsa-miR-583 | 355 | 303 | O19 | | CAAAGAGGAAGGUCCCAUUAC |
| hsa-miR-483-3p | 356 | 319 | O20 | | UCACUCCUCUCCUCCCGUCUU |
| hsa-miR-582-5p | 357 | 335 | O21 | | UUACAGUUGUUCAACCAGUUACU |
| hsa-miR-183-5p | 358 | 351 | O22 | | UAUGGCACUGGUAGAAUUCACU |
| hsa-miR-33b-5p | 359 | 367 | O23 | | GUGCAUUGCUGUUGCAUUGC |
| hsa-miR-193a-3p | 360 | 383 | O24 | | AACUGGCCUACAAAGUCCCAGU |
| hsa-miR-153-3p | 361 | 16 | P01 | | UUGCAUAGUCACAAAAGUGAUC |
| hsa-let-7e-5p | 362 | 32 | P02 | | UGAGGUAGGAGGUUGUAUAGUU |
| hsa-miR-409-3p | 363 | 48 | P03 | | GAAUGUUGCUCGGUGAACCCCU |
| hsa-miR-100-5p | 364 | 64 | P04 | | AACCCGUAGAUCCGAACUUGUG |
| hsa-miR-629-5p | 365 | 80 | P05 | | UGGGUUUACGUUGGGAGAACU |
| hsa-miR-484 | 366 | 96 | P06 | | UCAGGCUCAGUCCCCUCCCGAU |
| hsa-miR-429 | 367 | 112 | P07 | | UAAUACUGUCUGGUAAAACCGU |
| hsa-miR-30c-2-3p | 368 | 128 | P08 | | CUGGGAGAAGGCUGUUUACUCU |
| hsa-miR-518a-3p | 369 | 144 | P09 | | GAAAGCGCUUCCCUUUGCUGGA |
| hsa-miR-340-5p | 370 | 160 | P10 | | UUAUAAAGCAAUGAGACUGAUU |
| hsa-miR-508-3p | 371 | 176 | P11 | | UGAUUGUAGCCUUUUGGAGUAGA |
| hsa-miR-381-3p | 372 | 192 | P12 | | UAUACAAGGGCAAGCUCUCUGU |
| hsa-miR-148a-3p | 373 | 208 | P13 | | UCAGUGCACUACAGAACUUUGU |
| hsa-miR-146a-5p | 374 | 224 | P14 | | UGAGAACUGAAUUCCAUGGGUU |
| hsa-miR-139-5p | 375 | 240 | P15 | | UCUACAGUGCACGUGUCUCCAGU |
| hsa-miR-373-5p | 376 | 256 | P16 | | ACUCAAAAUGGGGGCGCUUUCC |
| hsa-miR-149-5p | 377 | 272 | P17 | | UCUGGCUCCGUGUCUUCACUCCC |
| hsa-miR-642a-5p | 378 | 288 | P18 | | GUCCCUCUCCAAAUGUGUCUUG |
| hsa-miR-31-5p | 379 | 304 | P19 | | AGGCAAGAUGCUGGCAUAGCU |
| hsa-miR-451a | 380 | 320 | P20 | | AAACCGUUACCAUUACUGAGUU |
| hsa-miR-620 | 381 | 336 | P21 | | AUGGAGAUAGAUAUAGAAAU |
| hsa-miR-27b-3p | 382 | 352 | P22 | | UUCACAGUGGCUAAGUUCUGC |
| hsa-miR-523-3p | 383 | 368 | P23 | | GAACGCGCUUCCCUAUAGAGGGU |
| hsa-miR-374a-5p | 384 | 384 | P24 | | UUAUAAUACAACCUGAUAAGUG |
|  |  |  |  | |  |
| **Panel II** |  |  |  | |  |
| hsa-miR-92a-1-5p | 1 | 1 | A01 | | AGGUUGGGAUCGGUUGCAAUGCU |
| hsa-miR-219a-1-3p | 2 | 17 | A02 | | AGAGUUGAGUCUGGACGUCCCG |
| hsa-miR-1913 | 3 | 33 | A03 | | UCUGCCCCCUCCGCUGCUGCCA |
| hsa-miR-1245a | 4 | 49 | A04 | | AAGUGAUCUAAAGGCCUACAU |
| hsa-miR-522-3p | 5 | 65 | A05 | | AAAAUGGUUCCCUUUAGAGUGU |
| hsa-miR-571 | 6 | 81 | A06 | | UGAGUUGGCCAUCUGAGUGAG |
| hsa-miR-323a-5p | 7 | 97 | A07 | | AGGUGGUCCGUGGCGCGUUCGC |
| hsa-miR-592 | 8 | 113 | A08 | | UUGUGUCAAUAUGCGAUGAUGU |
| hsa-miR-487a-3p | 9 | 129 | A09 | | AAUCAUACAGGGACAUCCAGUU |
| hsa-miR-1249 | 10 | 145 | A10 | | ACGCCCUUCCCCCCCUUCUUCA |
| hsa-miR-25-5p | 11 | 161 | A11 | | AGGCGGAGACUUGGGCAAUUG |
| hsa-miR-922 | 12 | 177 | A12 | | GCAGCAGAGAAUAGGACUACGUC |
| hsa-miR-124-5p | 13 | 193 | A13 | | CGUGUUCACAGCGGACCUUGAU |
| hsa-miR-1264 | 14 | 209 | A14 | | CAAGUCUUAUUUGAGCACCUGUU |
| hsa-miR-504-5p | 15 | 225 | A15 | | AGACCCUGGUCUGCACUCUAUC |
| hsa-miR-138-1-3p | 16 | 241 | A16 | | GCUACUUCACAACACCAGGGCC |
| hsa-miR-502-3p | 17 | 257 | A17 | | AAUGCACCUGGGCAAGGAUUCA |
| hsa-miR-490-5p | 18 | 273 | A18 | | CCAUGGAUCUCCAGGUGGGU |
| hsa-miR-567 | 19 | 289 | A19 | | AGUAUGUUCUUCCAGGACAGAAC |
| hsa-miR-18b-3p | 20 | 305 | A20 | | UGCCCUAAAUGCCCCUUCUGGC |
| hsa-miR-125a-3p | 21 | 321 | A21 | | ACAGGUGAGGUUCUUGGGAGCC |
| hsa-miR-653-5p | 22 | 337 | A22 | | GUGUUGAAACAAUCUCUACUG |
| hsa-miR-891b | 23 | 353 | A23 | | UGCAACUUACCUGAGUCAUUGA |
| hsa-miR-144-5p | 24 | 369 | A24 | | GGAUAUCAUCAUAUACUGUAAG |
| hsa-miR-1538 | 25 | 2 | B01 | | CGGCCCGGGCUGCUGCUGUUCCU |
| hsa-miR-384 | 26 | 18 | B02 | | AUUCCUAGAAAUUGUUCAUA |
| hsa-miR-196b-3p | 27 | 34 | B03 | | UCGACAGCACGACACUGCCUUC |
| hsa-miR-649 | 28 | 50 | B04 | | AAACCUGUGUUGUUCAAGAGUC |
| hsa-miR-143-5p | 29 | 66 | B05 | | GGUGCAGUGCUGCAUCUCUGGU |
| hsa-miR-1207-5p | 30 | 82 | B06 | | UGGCAGGGAGGCUGGGAGGGG |
| hsa-miR-943 | 31 | 98 | B07 | | CUGACUGUUGCCGUCCUCCAG |
| hsa-miR-675-3p | 32 | 114 | B08 | | CUGUAUGCCCUCACCGCUCA |
| hsa-miR-200b-5p | 33 | 130 | B09 | | CAUCUUACUGGGCAGCAUUGGA |
| hsa-miR-519e-5p | 34 | 146 | B10 | | UUCUCCAAAAGGGAGCACUUUC |
| hsa-miR-942-5p | 35 | 162 | B11 | | UCUUCUCUGUUUUGGCCAUGUG |
| hsa-miR-450b-3p | 36 | 178 | B12 | | UUGGGAUCAUUUUGCAUCCAUA |
| hsa-miR-553 | 37 | 194 | B13 | | AAAACGGUGAGAUUUUGUUUU |
| hsa-miR-605-5p | 38 | 210 | B14 | | UAAAUCCCAUGGUGCCUUCUCCU |
| hsa-miR-24-2-5p | 39 | 226 | B15 | | UGCCUACUGAGCUGAAACACAG |
| hsa-miR-23a-5p | 40 | 242 | B16 | | GGGGUUCCUGGGGAUGGGAUUU |
| hsa-miR-27b-5p | 41 | 258 | B17 | | AGAGCUUAGCUGAUUGGUGAAC |
| hsa-miR-759 | 42 | 274 | B18 | | GCAGAGUGCAAACAAUUUUGAC |
| hsa-miR-770-5p | 43 | 290 | B19 | | UCCAGUACCACGUGUCAGGGCCA |
| hsa-miR-585-3p | 44 | 306 | B20 | | UGGGCGUAUCUGUAUGCUA |
| hsa-miR-376a-5p | 45 | 322 | B21 | | GUAGAUUCUCCUUCUAUGAGUA |
| hsa-miR-507 | 46 | 338 | B22 | | UUUUGCACCUUUUGGAGUGAA |
| hsa-miR-520b | 47 | 354 | B23 | | AAAGUGCUUCCUUUUAGAGGG |
| hsa-miR-302f | 48 | 370 | B24 | | UAAUUGCUUCCAUGUUU |
| hsa-miR-28-3p | 49 | 3 | C01 | | CACUAGAUUGUGAGCUCCUGGA |
| hsa-miR-875-5p | 50 | 19 | C02 | | UAUACCUCAGUUUUAUCAGGUG |
| hsa-miR-219a-2-3p | 51 | 35 | C03 | | AGAAUUGUGGCUGGACAUCUGU |
| hsa-miR-1183 | 52 | 51 | C04 | | CACUGUAGGUGAUGGUGAGAGUGGGCA |
| hsa-miR-758-3p | 53 | 67 | C05 | | UUUGUGACCUGGUCCACUAACC |
| hsa-miR-1244 | 54 | 83 | C06 | | AAGUAGUUGGUUUGUAUGAGAUGGUU |
| hsa-miR-566 | 55 | 99 | C07 | | GGGCGCCUGUGAUCCCAAC |
| hsa-miR-1256 | 56 | 115 | C08 | | AGGCAUUGACUUCUCACUAGCU |
| hsa-miR-516a-3p | 57 | 131 | C09 | | UGCUUCCUUUCAGAGGGU |
| hsa-miR-548c-5p | 58 | 147 | C10 | | AAAAGUAAUUGCGGUUUUUGCC |
| hsa-miR-496 | 59 | 163 | C11 | | UGAGUAUUACAUGGCCAAUCUC |
| hsa-miR-876-3p | 60 | 179 | C12 | | UGGUGGUUUACAAAGUAAUUCA |
| hsa-miR-532-3p | 61 | 195 | C13 | | CCUCCCACACCCAAGGCUUGCA |
| hsa-miR-654-3p | 62 | 211 | C14 | | UAUGUCUGCUGACCAUCACCUU |
| hsa-miR-659-3p | 63 | 227 | C15 | | CUUGGUUCAGGGAGGGUCCCCA |
| hsa-miR-135b-3p | 64 | 243 | C16 | | AUGUAGGGCUAAAAGCCAUGGG |
| hsa-miR-641 | 65 | 259 | C17 | | AAAGACAUAGGAUAGAGUCACCUC |
| hsa-miR-2113 | 66 | 275 | C18 | | AUUUGUGCUUGGCUCUGUCAC |
| hsa-miR-1254 | 67 | 291 | C19 | | AGCCUGGAAGCUGGAGCCUGCAGU |
| hsa-miR-661 | 68 | 307 | C20 | | UGCCUGGGUCUCUGGCCUGCGCGU |
| hsa-miR-892a | 69 | 323 | C21 | | CACUGUGUCCUUUCUGCGUAG |
| hsa-miR-10b-3p | 70 | 339 | C22 | | ACAGAUUCGAUUCUAGGGGAAU |
| hsa-miR-122-3p | 71 | 355 | C23 | | AACGCCAUUAUCACACUAAAUA |
| hsa-miR-100-3p | 72 | 371 | C24 | | CAAGCUUGUAUCUAUAGGUAUG |
| hsa-miR-769-3p | 73 | 4 | D01 | | CUGGGAUCUCCGGGGUCUUGGUU |
| hsa-miR-300 | 74 | 20 | D02 | | UAUACAAGGGCAGACUCUCUCU |
| hsa-miR-518e-5p | 75 | 36 | D03 | | CUCUAGAGGGAAGCGCUUUCUG |
| hsa-miR-489-3p | 76 | 52 | D04 | | GUGACAUCACAUAUACGGCAGC |
| hsa-miR-937-3p | 77 | 68 | D05 | | AUCCGCGCUCUGACUCUCUGCC |
| hsa-miR-381-5p | 78 | 84 | D06 | | AGCGAGGUUGCCCUUUGUAUAU |
| hsa-miR-640 | 79 | 100 | D07 | | AUGAUCCAGGAACCUGCCUCU |
| hsa-miR-148b-5p | 80 | 116 | D08 | | AAGUUCUGUUAUACACUCAGGC |
| hsa-miR-29c-5p | 81 | 132 | D09 | | UGACCGAUUUCUCCUGGUGUUC |
| hsa-miR-499a-3p | 82 | 148 | D10 | | AACAUCACAGCAAGUCUGUGCU |
| hsa-let-7f-1-3p | 83 | 164 | D11 | | CUAUACAAUCUAUUGCCUUCCC |
| hsa-miR-382-3p | 84 | 180 | D12 | | AAUCAUUCACGGACAACACUU |
| hsa-miR-609 | 85 | 196 | D13 | | AGGGUGUUUCUCUCAUCUCU |
| hsa-miR-10a-3p | 86 | 212 | D14 | | CAAAUUCGUAUCUAGGGGAAUA |
| hsa-miR-106a-3p | 87 | 228 | D15 | | CUGCAAUGUAAGCACUUCUUAC |
| hsa-let-7e-3p | 88 | 244 | D16 | | CUAUACGGCCUCCUAGCUUUCC |
| hsa-miR-580-3p | 89 | 260 | D17 | | UUGAGAAUGAUGAAUCAUUAGG |
| hsa-miR-761 | 90 | 276 | D18 | | GCAGCAGGGUGAAACUGACACA |
| hsa-miR-643 | 91 | 292 | D19 | | ACUUGUAUGCUAGCUCAGGUAG |
| hsa-miR-618 | 92 | 308 | D20 | | AAACUCUACUUGUCCUUCUGAGU |
| hsa-miR-221-5p | 93 | 324 | D21 | | ACCUGGCAUACAAUGUAGAUUU |
| hsa-miR-513b-5p | 94 | 340 | D22 | | UUCACAAGGAGGUGUCAUUUAU |
| hsa-miR-411-3p | 95 | 356 | D23 | | UAUGUAACACGGUCCACUAACC |
| hsa-miR-19a-5p | 96 | 372 | D24 | | AGUUUUGCAUAGUUGCACUACA |
| hsa-miR-338-5p | 97 | 5 | E01 | | AACAAUAUCCUGGUGCUGAGUG |
| hsa-miR-1914-3p | 98 | 21 | E02 | | GGAGGGGUCCCGCACUGGGAGG |
| hsa-miR-323b-5p | 99 | 37 | E03 | | AGGUUGUCCGUGGUGAGUUCGCA |
| hsa-miR-548i | 100 | 53 | E04 | | AAAAGUAAUUGCGGAUUUUGCC |
| hsa-miR-541-3p | 101 | 69 | E05 | | UGGUGGGCACAGAAUCUGGACU |
| hsa-miR-1272 | 102 | 85 | E06 | | GAUGAUGAUGGCAGCAAAUUCUGAAA |
| hsa-miR-1205 | 103 | 101 | E07 | | UCUGCAGGGUUUGCUUUGAG |
| hsa-miR-544a | 104 | 117 | E08 | | AUUCUGCAUUUUUAGCAAGUUC |
| hsa-miR-431-3p | 105 | 133 | E09 | | CAGGUCGUCUUGCAGGGCUUCU |
| hsa-miR-621 | 106 | 149 | E10 | | GGCUAGCAACAGCGCUUACCU |
| hsa-miR-556-5p | 107 | 165 | E11 | | GAUGAGCUCAUUGUAAUAUGAG |
| hsa-miR-1267 | 108 | 181 | E12 | | CCUGUUGAAGUGUAAUCCCCA |
| hsa-miR-141-5p | 109 | 197 | E13 | | CAUCUUCCAGUACAGUGUUGGA |
| hsa-miR-1269a | 110 | 213 | E14 | | CUGGACUGAGCCGUGCUACUGG |
| hsa-miR-501-3p | 111 | 229 | E15 | | AAUGCACCCGGGCAAGGAUUCU |
| hsa-miR-15b-3p | 112 | 245 | E16 | | CGAAUCAUUAUUUGCUGCUCUA |
| hsa-miR-146b-3p | 113 | 261 | E17 | | UGCCCUGUGGACUCAGUUCUGG |
| hsa-miR-222-5p | 114 | 277 | E18 | | CUCAGUAGCCAGUGUAGAUCCU |
| hsa-miR-601 | 115 | 293 | E19 | | UGGUCUAGGAUUGUUGGAGGAG |
| hsa-miR-924 | 116 | 309 | E20 | | AGAGUCUUGUGAUGUCUUGC |
| hsa-miR-29a-5p | 117 | 325 | E21 | | ACUGAUUUCUUUUGGUGUUCAG |
| hsa-let-7a-2-3p | 118 | 341 | E22 | | CUGUACAGCCUCCUAGCUUUCC |
| hsa-miR-520f-3p | 119 | 357 | E23 | | AAGUGCUUCCUUUUAGAGGGUU |
| hsa-miR-101-5p | 120 | 373 | E24 | | CAGUUAUCACAGUGCUGAUGCU |
| hsa-miR-520a-3p | 121 | 6 | F01 | | AAAGUGCUUCCCUUUGGACUGU |
| hsa-miR-548m | 122 | 22 | F02 | | CAAAGGUAUUUGUGGUUUUUG |
| hsa-miR-517-5p | 123 | 38 | F03 | | CCUCUAGAUGGAAGCACUGUCU |
| hsa-miR-448 | 124 | 54 | F04 | | UUGCAUAUGUAGGAUGUCCCAU |
| hsa-miR-1296-5p | 125 | 70 | F05 | | UUAGGGCCCUGGCUCCAUCUCC |
| hsa-miR-1537-3p | 126 | 86 | F06 | | AAAACCGUCUAGUUACAGUUGU |
| hsa-miR-920 | 127 | 102 | F07 | | GGGGAGCUGUGGAAGCAGUA |
| hsa-miR-1247-5p | 128 | 118 | F08 | | ACCCGUCCCGUUCGUCCCCGGA |
| hsa-miR-19b-2-5p | 129 | 134 | F09 | | AGUUUUGCAGGUUUGCAUUUCA |
| hsa-miR-558 | 130 | 150 | F10 | | UGAGCUGCUGUACCAAAAU |
| hsa-miR-106b-3p | 131 | 166 | F11 | | CCGCACUGUGGGUACUUGCUGC |
| hsa-miR-1258 | 132 | 182 | F12 | | AGUUAGGAUUAGGUCGUGGAA |
| hsa-miR-619-3p | 133 | 198 | F13 | | GACCUGGACAUGUUUGUGCCCAGU |
| hsa-miR-208a-3p | 134 | 214 | F14 | | AUAAGACGAGCAAAAAGCUUGU |
| hsa-miR-17-3p | 135 | 230 | F15 | | ACUGCAGUGAAGGCACUUGUAG |
| hsa-miR-136-3p | 136 | 246 | F16 | | CAUCAUCGUCUCAAAUGAGUCU |
| hsa-miR-877-3p | 137 | 262 | F17 | | UCCUCUUCUCCCUCCUCCCAG |
| hsa-miR-935 | 138 | 278 | F18 | | CCAGUUACCGCUUCCGCUACCGC |
| hsa-miR-224-3p | 139 | 294 | F19 | | AAAAUGGUGCCCUAGUGACUACA |
| hsa-miR-624-3p | 140 | 310 | F20 | | CACAAGGUAUUGGUAUUACCU |
| hsa-miR-767-5p | 141 | 326 | F21 | | UGCACCAUGGUUGUCUGAGCAUG |
| hsa-miR-559 | 142 | 342 | F22 | | UAAAGUAAAUAUGCACCAAAA |
| hsa-miR-449b-3p | 143 | 358 | F23 | | CAGCCACAACUACCCUGCCACU |
| hsa-miR-205-3p | 144 | 374 | F24 | | GAUUUCAGUGGAGUGAAGUUC |
| hsa-miR-604 | 145 | 7 | G01 | | AGGCUGCGGAAUUCAGGAC |
| hsa-miR-130b-5p | 146 | 23 | G02 | | ACUCUUUCCCUGUUGCACUAC |
| hsa-miR-149-3p | 147 | 39 | G03 | | AGGGAGGGACGGGGGCUGUGC |
| hsa-miR-1271-5p | 148 | 55 | G04 | | CUUGGCACCUAGCAAGCACUCA |
| hsa-miR-520h | 149 | 71 | G05 | | ACAAAGUGCUUCCCUUUAGAGU |
| hsa-miR-769-5p | 150 | 87 | G06 | | UGAGACCUCUGGGUUCUGAGCU |
| hsa-miR-612 | 151 | 103 | G07 | | GCUGGGCAGGGCUUCUGAGCUCCUU |
| hsa-miR-1237-3p | 152 | 119 | G08 | | UCCUUCUGCUCCGUCCCCCAG |
| hsa-miR-1908-5p | 153 | 135 | G09 | | CGGCGGGGACGGCGAUUGGUC |
| hsa-miR-1260a | 154 | 151 | G10 | | AUCCCACCUCUGCCACCA |
| hsa-miR-182-3p | 155 | 167 | G11 | | UGGUUCUAGACUUGCCAACUA |
| hsa-miR-365b-5p | 156 | 183 | G12 | | AGGGACUUUCAGGGGCAGCUGU |
| hsa-miR-508-5p | 157 | 199 | G13 | | UACUCCAGAGGGCGUCACUCAUG |
| hsa-miR-671-3p | 158 | 215 | G14 | | UCCGGUUCUCAGGGCUCCACC |
| hsa-miR-941 | 159 | 231 | G15 | | CACCCGGCUGUGUGCACAUGUGC |
| hsa-miR-23b-5p | 160 | 247 | G16 | | UGGGUUCCUGGCAUGCUGAUUU |
| hsa-miR-591 | 161 | 263 | G17 | | AGACCAUGGGUUCUCAUUGU |
| hsa-miR-26b-3p | 162 | 279 | G18 | | CCUGUUCUCCAUUACUUGGCUC |
| hsa-miR-519b-3p | 163 | 295 | G19 | | AAAGUGCAUCCUUUUAGAGGUU |
| hsa-miR-30d-3p | 164 | 311 | G20 | | CUUUCAGUCAGAUGUUUGCUGC |
| hsa-miR-518d-5p | 165 | 327 | G21 | | CUCUAGAGGGAAGCACUUUCUG |
| hsa-miR-212-5p | 166 | 343 | G22 | | ACCUUGGCUCUAGACUGCUUACU |
| hsa-miR-520e | 167 | 359 | G23 | | AAAGUGCUUCCUUUUUGAGGG |
| hsa-miR-646 | 168 | 375 | G24 | | AAGCAGCUGCCUCUGAGGC |
| hsa-miR-519e-3p | 169 | 8 | H01 | | AAGUGCCUCCUUUUAGAGUGUU |
| hsa-miR-626 | 170 | 24 | H02 | | AGCUGUCUGAAAAUGUCUU |
| hsa-miR-26a-1-3p | 171 | 40 | H03 | | CCUAUUCUUGGUUACUUGCACG |
| hsa-miR-190b | 172 | 56 | H04 | | UGAUAUGUUUGAUAUUGGGUU |
| hsa-miR-1471 | 173 | 72 | H05 | | GCCCGCGUGUGGAGCCAGGUGU |
| hsa-miR-548l | 174 | 88 | H06 | | AAAAGUAUUUGCGGGUUUUGUC |
| hsa-miR-586 | 175 | 104 | H07 | | UAUGCAUUGUAUUUUUAGGUCC |
| hsa-miR-103b | 176 | 120 | H08 | | UCAUAGCCCUGUACAAUGCUGCU |
| hsa-miR-488-5p | 177 | 136 | H09 | | CCCAGAUAAUGGCACUCUCAA |
| hsa-miR-129-1-3p | 178 | 152 | H10 | | AAGCCCUUACCCCAAAAAGUAU |
| hsa-miR-192-3p | 179 | 168 | H11 | | CUGCCAAUUCCAUAGGUCACAG |
| hsa-miR-632 | 180 | 184 | H12 | | GUGUCUGCUUCCUGUGGGA |
| hsa-miR-181a-2-3p | 181 | 200 | H13 | | ACCACUGACCGUUGACUGUACC |
| hsa-miR-1909-3p | 182 | 216 | H14 | | CGCAGGGGCCGGGUGCUCACCG |
| hsa-miR-573 | 183 | 232 | H15 | | CUGAAGUGAUGUGUAACUGAUCAG |
| hsa-miR-302d-5p | 184 | 248 | H16 | | ACUUUAACAUGGAGGCACUUGC |
| hsa-miR-194-3p | 185 | 264 | H17 | | CCAGUGGGGCUGCUGUUAUCUG |
| hsa-miR-302b-5p | 186 | 280 | H18 | | ACUUUAACAUGGAAGUGCUUUC |
| hsa-miR-551b-5p | 187 | 296 | H19 | | GAAAUCAAGCGUGGGUGAGACC |
| hsa-miR-635 | 188 | 312 | H20 | | ACUUGGGCACUGAAACAAUGUCC |
| hsa-miR-518d-3p | 189 | 328 | H21 | | CAAAGCGCUUCCCUUUGGAGC |
| hsa-miR-569 | 190 | 344 | H22 | | AGUUAAUGAAUCCUGGAAAGU |
| hsa-miR-125b-1-3p | 191 | 360 | H23 | | ACGGGUUAGGCUCUUGGGAGCU |
| hsa-miR-218-2-3p | 192 | 376 | H24 | | CAUGGUUCUGUCAAGCACCGCG |
| hsa-miR-519c-3p | 193 | 9 | I01 | | AAAGUGCAUCUUUUUAGAGGAU |
| hsa-miR-554 | 194 | 25 | I02 | | GCUAGUCCUGACUCAGCCAGU |
| hsa-miR-938 | 195 | 41 | I03 | | UGCCCUUAAAGGUGAACCCAGU |
| hsa-miR-1243 | 196 | 57 | I04 | | AACUGGAUCAAUUAUAGGAGUG |
| hsa-miR-708-3p | 197 | 73 | I05 | | CAACUAGACUGUGAGCUUCUAG |
| hsa-miR-1185-5p | 198 | 89 | I06 | | AGAGGAUACCCUUUGUAUGUU |
| hsa-miR-512-3p | 199 | 105 | I07 | | AAGUGCUGUCAUAGCUGAGGUC |
| hsa-miR-587 | 200 | 121 | I08 | | UUUCCAUAGGUGAUGAGUCAC |
| hsa-miR-603 | 201 | 137 | I09 | | CACACACUGCAAUUACUUUUGC |
| hsa-miR-1184 | 202 | 153 | I10 | | CCUGCAGCGACUUGAUGGCUUCC |
| hsa-miR-20a-3p | 203 | 169 | I11 | | ACUGCAUUAUGAGCACUUAAAG |
| hsa-miR-588 | 204 | 185 | I12 | | UUGGCCACAAUGGGUUAGAAC |
| hsa-miR-455-3p | 205 | 201 | I13 | | GCAGUCCAUGGGCAUAUACAC |
| hsa-miR-582-3p | 206 | 217 | I14 | | UAACUGGUUGAACAACUGAACC |
| hsa-miR-409-5p | 207 | 233 | I15 | | AGGUUACCCGAGCAACUUUGCAU |
| hsa-miR-452-3p | 208 | 249 | I16 | | CUCAUCUGCAAAGAAGUAAGUG |
| hsa-miR-19b-1-5p | 209 | 265 | I17 | | AGUUUUGCAGGUUUGCAUCCAGC |
| hsa-miR-610 | 210 | 281 | I18 | | UGAGCUAAAUGUGUGCUGGGA |
| hsa-miR-511-5p | 211 | 297 | I19 | | GUGUCUUUUGCUCUGCAGUCA |
| hsa-miR-200c-5p | 212 | 313 | I20 | | CGUCUUACCCAGCAGUGUUUGG |
| hsa-let-7a-3p | 213 | 329 | I21 | | CUAUACAAUCUACUGUCUUUC |
| hsa-miR-135a-3p | 214 | 345 | I22 | | UAUAGGGAUUGGAGCCGUGGCG |
| hsa-miR-520a-5p | 215 | 361 | I23 | | CUCCAGAGGGAAGUACUUUCU |
| hsa-miR-1468-5p | 216 | 377 | I24 | | CUCCGUUUGCCUGUUUCGCUG |
| hsa-miR-628-5p | 217 | 10 | J01 | | AUGCUGACAUAUUUACUAGAGG |
| hsa-miR-552-3p | 218 | 26 | J02 | | AACAGGUGACUGGUUAGACAA |
| hsa-miR-145-3p | 219 | 42 | J03 | | GGAUUCCUGGAAAUACUGUUCU |
| Blank (H2O) | 220 | 58 | J04 | |  |
| hsa-miR-378a-5p | 221 | 74 | J05 | | CUCCUGACUCCAGGUCCUGUGU |
| hsa-miR-7-1-3p | 222 | 90 | J06 | | CAACAAAUCACAGUCUGCCAUA |
| hsa-miR-181c-3p | 223 | 106 | J07 | | AACCAUCGACCGUUGAGUGGAC |
| hsa-miR-195-3p | 224 | 122 | J08 | | CCAAUAUUGGCUGUGCUGCUCC |
| hsa-miR-578 | 225 | 138 | J09 | | CUUCUUGUGCUCUAGGAUUGU |
| hsa-miR-505-5p | 226 | 154 | J10 | | GGGAGCCAGGAAGUAUUGAUGU |
| hsa-miR-875-3p | 227 | 170 | J11 | | CCUGGAAACACUGAGGUUGUG |
| hsa-miR-450b-5p | 228 | 186 | J12 | | UUUUGCAAUAUGUUCCUGAAUA |
| hsa-miR-876-5p | 229 | 202 | J13 | | UGGAUUUCUUUGUGAAUCACCA |
| hsa-miR-362-3p | 230 | 218 | J14 | | AACACACCUAUUCAAGGAUUCA |
| hsa-miR-624-5p | 231 | 234 | J15 | | UAGUACCAGUACCUUGUGUUCA |
| hsa-miR-27a-5p | 232 | 250 | J16 | | AGGGCUUAGCUGCUUGUGAGCA |
| hsa-miR-744-3p | 233 | 266 | J17 | | CUGUUGCCACUAACCUCAACCU |
| hsa-miR-139-3p | 234 | 282 | J18 | | UGGAGACGCGGCCCUGUUGGAGU |
| hsa-miR-138-2-3p | 235 | 298 | J19 | | GCUAUUUCACGACACCAGGGUU |
| hsa-miR-655-3p | 236 | 314 | J20 | | AUAAUACAUGGUUAACCUCUUU |
| hsa-miR-99b-3p | 237 | 330 | J21 | | CAAGCUCGUGUCUGUGGGUCCG |
| hsa-miR-581 | 238 | 346 | J22 | | UCUUGUGUUCUCUAGAUCAGU |
| hsa-miR-191-3p | 239 | 362 | J23 | | GCUGCGCUUGGAUUUCGUCCCC |
| hsa-miR-32-3p | 240 | 378 | J24 | | CAAUUUAGUGUGUGUGAUAUUU |
| hsa-miR-1204 | 241 | 11 | K01 | | UCGUGGCCUGGUCUCCAUUAU |
| hsa-miR-548j-5p | 242 | 27 | K02 | | AAAAGUAAUUGCGGUCUUUGGU |
| hsa-miR-555 | 243 | 43 | K03 | | AGGGUAAGCUGAACCUCUGAU |
| hsa-miR-1224-3p | 244 | 59 | K04 | | CCCCACCUCCUCUCUCCUCAG |
| hsa-miR-1539 | 245 | 75 | K05 | | UCCUGCGCGUCCCAGAUGCCC |
| hsa-miR-663b | 246 | 91 | K06 | | GGUGGCCCGGCCGUGCCUGAGG |
| UniSp3 IPC | 247 | 107 | K07 | |  |
| hsa-miR-1248 | 248 | 123 | K08 | | ACCUUCUUGUAUAAGCACUGUGCUAAA |
| hsa-miR-889-3p | 249 | 139 | K09 | | UUAAUAUCGGACAACCAUUGU |
| hsa-miR-1227-3p | 250 | 155 | K10 | | CGUGCCACCCUUUUCCCCAG |
| hsa-miR-548h-5p | 251 | 171 | K11 | | AAAAGUAAUCGCGGUUUUUGUC |
| hsa-miR-1255b-5p | 252 | 187 | K12 | | CGGAUGAGCAAAGAAAGUGGUU |
| hsa-miR-330-5p | 253 | 203 | K13 | | UCUCUGGGCCUGUGUCUUAGGC |
| hsa-miR-1238-3p | 254 | 219 | K14 | | CUUCCUCGUCUGUCUGCCCC |
| hsa-miR-188-3p | 255 | 235 | K15 | | CUCCCACAUGCAGGGUUUGCA |
| hsa-miR-589-3p | 256 | 251 | K16 | | UCAGAACAAAUGCCGGUUCCCAGA |
| hsa-miR-125b-2-3p | 257 | 267 | K17 | | UCACAAGUCAGGCUCUUGGGAC |
| hsa-miR-16-2-3p | 258 | 283 | K18 | | CCAAUAUUACUGUGCUGCUUUA |
| hsa-miR-515-5p | 259 | 299 | K19 | | UUCUCCAAAAGAAAGCACUUUCUG |
| hsa-miR-340-3p | 260 | 315 | K20 | | UCCGUCUCAGUUACUUUAUAGC |
| hsa-miR-513a-3p | 261 | 331 | K21 | | UAAAUUUCACCUUUCUGAGAAGG |
| hsa-miR-34a-3p | 262 | 347 | K22 | | CAAUCAGCAAGUAUACUGCCCU |
| hsa-miR-342-5p | 263 | 363 | K23 | | AGGGGUGCUAUCUGUGAUUGA |
| hsa-miR-639 | 264 | 379 | K24 | | AUCGCUGCGGUUGCGAGCGCUGU |
| hsa-let-7i-3p | 265 | 12 | L01 | | CUGCGCAAGCUACUGCCUUGCU |
| hsa-miR-543 | 266 | 28 | L02 | | AAACAUUCGCGGUGCACUUCUU |
| hsa-miR-645 | 267 | 44 | L03 | | UCUAGGCUGGUACUGCUGA |
| hsa-miR-548d-5p | 268 | 60 | L04 | | AAAAGUAAUUGUGGUUUUUGCC |
| hsa-miR-33a-3p | 269 | 76 | L05 | | CAAUGUUUCCACAGUGCAUCAC |
| hsa-miR-664a-3p | 270 | 92 | L06 | | UAUUCAUUUAUCCCCAGCCUACA |
| UniSp3 IPC | 271 | 108 | L07 | |  |
| UniSp3 IPC | 272 | 124 | L08 | |  |
| hsa-miR-379-3p | 273 | 140 | L09 | | UAUGUAACAUGGUCCACUAACU |
| hsa-miR-556-3p | 274 | 156 | L10 | | AUAUUACCAUUAGCUCAUCUUU |
| hsa-miR-614 | 275 | 172 | L11 | | GAACGCCUGUUCUUGCCAGGUGG |
| hsa-miR-616-5p | 276 | 188 | L12 | | ACUCAAAACCCUUCAGUGACUU |
| hsa-miR-93-3p | 277 | 204 | L13 | | ACUGCUGAGCUAGCACUUCCCG |
| hsa-miR-1972 | 278 | 220 | L14 | | UCAGGCCAGGCACAGUGGCUCA |
| hsa-miR-616-3p | 279 | 236 | L15 | | AGUCAUUGGAGGGUUUGAGCAG |
| hsa-miR-369-3p | 280 | 252 | L16 | | AAUAAUACAUGGUUGAUCUUU |
| hsa-miR-2110 | 281 | 268 | L17 | | UUGGGGAAACGGCCGCUGAGUG |
| hsa-miR-548a-3p | 282 | 284 | L18 | | CAAAACUGGCAAUUACUUUUGC |
| hsa-miR-634 | 283 | 300 | L19 | | AACCAGCACCCCAACUUUGGAC |
| hsa-miR-320c | 284 | 316 | L20 | | AAAAGCUGGGUUGAGAGGGU |
| hsa-miR-636 | 285 | 332 | L21 | | UGUGCUUGCUCGUCCCGCCCGCA |
| hsa-miR-606 | 286 | 348 | L22 | | AAACUACUGAAAAUCAAAGAU |
| hsa-miR-208b-3p | 287 | 364 | L23 | | AUAAGACGAACAAAAGGUUUGU |
| hsa-miR-367-5p | 288 | 380 | L24 | | ACUGUUGCUAAUAUGCAACUCU |
| hsa-miR-520d-3p | 289 | 13 | M01 | | AAAGUGCUUCUCUUUGGUGGGU |
| hsa-miR-1265 | 290 | 29 | M02 | | CAGGAUGUGGUCAAGUGUUGUU |
| hsa-miR-1203 | 291 | 45 | M03 | | CCCGGAGCCAGGAUGCAGCUC |
| hsa-miR-548k | 292 | 61 | M04 | | AAAAGUACUUGCGGAUUUUGCU |
| hsa-miR-548a-5p | 293 | 77 | M05 | | AAAAGUAAUUGCGAGUUUUACC |
| hsa-miR-1253 | 294 | 93 | M06 | | AGAGAAGAAGAUCAGCCUGCA |
| hsa-miR-615-5p | 295 | 109 | M07 | | GGGGGUCCCCGGUGCUCGGAUC |
| hsa-miR-607 | 296 | 125 | M08 | | GUUCAAAUCCAGAUCUAUAAC |
| hsa-miR-1208 | 297 | 141 | M09 | | UCACUGUUCAGACAGGCGGA |
| hsa-miR-302e | 298 | 157 | M10 | | UAAGUGCUUCCAUGCUU |
| hsa-miR-1206 | 299 | 173 | M11 | | UGUUCAUGUAGAUGUUUAAGC |
| hsa-miR-1270 | 300 | 189 | M12 | | CUGGAGAUAUGGAAGAGCUGUGU |
| hsa-miR-525-3p | 301 | 205 | M13 | | GAAGGCGCUUCCCUUUAGAGCG |
| hsa-miR-1200 | 302 | 221 | M14 | | CUCCUGAGCCAUUCUGAGCCUC |
| hsa-miR-1911-5p | 303 | 237 | M15 | | UGAGUACCGCCAUGUCUGUUGGG |
| hsa-miR-33b-3p | 304 | 253 | M16 | | CAGUGCCUCGGCAGUGCAGCCC |
| hsa-miR-223-5p | 305 | 269 | M17 | | CGUGUAUUUGACAAGCUGAGUU |
| hsa-miR-34b-5p | 306 | 285 | M18 | | UAGGCAGUGUCAUUAGCUGAUUG |
| hsa-miR-888-3p | 307 | 301 | M19 | | GACUGACACCUCUUUGGGUGAA |
| hsa-miR-424-3p | 308 | 317 | M20 | | CAAAACGUGAGGCGCUGCUAU |
| hsa-miR-339-3p | 309 | 333 | M21 | | UGAGCGCCUCGACGACAGAGCCG |
| hsa-miR-380-5p | 310 | 349 | M22 | | UGGUUGACCAUAGAACAUGCGC |
| hsa-miR-647 | 311 | 365 | M23 | | GUGGCUGCACUCACUUCCUUC |
| hsa-miR-518f-5p | 312 | 381 | M24 | | CUCUAGAGGGAAGCACUUUCUC |
| hsa-miR-92b-5p | 313 | 14 | N01 | | AGGGACGGGACGCGGUGCAGUG |
| hsa-miR-551a | 314 | 30 | N02 | | GCGACCCACUCUUGGUUUCCA |
| hsa-miR-146a-3p | 315 | 46 | N03 | | CCUCUGAAAUUCAGUUCUUCAG |
| hsa-miR-218-1-3p | 316 | 62 | N04 | | AUGGUUCCGUCAAGCACCAUGG |
| hsa-miR-593-5p | 317 | 78 | N05 | | AGGCACCAGCCAGGCAUUGCUCAGC |
| hsa-miR-561-3p | 318 | 94 | N06 | | CAAAGUUUAAGAUCCUUGAAGU |
| hsa-miR-767-3p | 319 | 110 | N07 | | UCUGCUCAUACCCCAUGGUUUCU |
| hsa-miR-526b-3p | 320 | 126 | N08 | | GAAAGUGCUUCCUUUUAGAGGC |
| hsa-miR-24-1-5p | 321 | 142 | N09 | | UGCCUACUGAGCUGAUAUCAGU |
| hsa-let-7b-3p | 322 | 158 | N10 | | CUAUACAACCUACUGCCUUCCC |
| hsa-miR-193b-5p | 323 | 174 | N11 | | CGGGGUUUUGAGGGCGAGAUGA |
| hsa-miR-335-3p | 324 | 190 | N12 | | UUUUUCAUUAUUGCUCCUGACC |
| hsa-miR-541-5p | 325 | 206 | N13 | | AAAGGAUUCUGCUGUCGGUCCCACU |
| hsa-miR-30c-1-3p | 326 | 222 | N14 | | CUGGGAGAGGGUUGUUUACUCC |
| hsa-miR-629-3p | 327 | 238 | N15 | | GUUCUCCCAACGUAAGCCCAGC |
| hsa-miR-377-5p | 328 | 254 | N16 | | AGAGGUUGCCCUUGGUGAAUUC |
| hsa-miR-630 | 329 | 270 | N17 | | AGUAUUCUGUACCAGGGAAGGU |
| hsa-miR-548d-3p | 330 | 286 | N18 | | CAAAAACCACAGUUUCUUUUGC |
| hsa-miR-885-3p | 331 | 302 | N19 | | AGGCAGCGGGGUGUAGUGGAUA |
| hsa-miR-320d | 332 | 318 | N20 | | AAAAGCUGGGUUGAGAGGA |
| hsa-miR-2053 | 333 | 334 | N21 | | GUGUUAAUUAAACCUCUAUUUAC |
| hsa-miR-675-5p | 334 | 350 | N22 | | UGGUGCGGAGAGGGCCCACAGUG |
| hsa-miR-1252-5p | 335 | 366 | N23 | | AGAAGGAAAUUGAAUUCAUUUA |
| hsa-miR-548e-3p | 336 | 382 | N24 | | AAAAACUGAGACUACUUUUGCA |
| hsa-miR-1914-5p | 337 | 15 | O01 | | CCCUGUGCCCGGCCCACUUCUG |
| hsa-miR-513c-5p | 338 | 31 | O02 | | UUCUCAAGGAGGUGUCGUUUAU |
| hsa-miR-331-5p | 339 | 47 | O03 | | CUAGGUAUGGUCCCAGGGAUCC |
| hsa-miR-1182 | 340 | 63 | O04 | | GAGGGUCUUGGGAGGGAUGUGAC |
| hsa-miR-611 | 341 | 79 | O05 | | GCGAGGACCCCUCGGGGUCUGAC |
| hsa-miR-1181 | 342 | 95 | O06 | | CCGUCGCCGCCACCCGAGCCG |
| hsa-miR-638 | 343 | 111 | O07 | | AGGGAUCGCGGGCGGGUGGCGGCCU |
| hsa-miR-515-3p | 344 | 127 | O08 | | GAGUGCCUUCUUUUGGAGCGUU |
| hsa-miR-650 | 345 | 143 | O09 | | AGGAGGCAGCGCUCUCAGGAC |
| hsa-miR-1178-3p | 346 | 159 | O10 | | UUGCUCACUGUUCUUCCCUAG |
| hsa-miR-600 | 347 | 175 | O11 | | ACUUACAGACAAGAGCCUUGCUC |
| hsa-miR-599 | 348 | 191 | O12 | | GUUGUGUCAGUUUAUCAAAC |
| hsa-miR-520g-3p | 349 | 207 | O13 | | ACAAAGUGCUUCCCUUUAGAGUGU |
| hsa-miR-564 | 350 | 223 | O14 | | AGGCACGGUGUCAGCAGGC |
| hsa-miR-132-5p | 351 | 239 | O15 | | ACCGUGGCUUUCGAUUGUUACU |
| hsa-miR-577 | 352 | 255 | O16 | | UAGAUAAAAUAUUGGUACCUG |
| hsa-miR-1911-3p | 353 | 271 | O17 | | CACCAGGCAUUGUGGUCUCC |
| hsa-let-7f-2-3p | 354 | 287 | O18 | | CUAUACAGUCUACUGUCUUUCC |
| hsa-miR-155-3p | 355 | 303 | O19 | | CUCCUACAUAUUAGCAUUAACA |
| hsa-miR-105-3p | 356 | 319 | O20 | | ACGGAUGUUUGAGCAUGUGCUA |
| hsa-miR-486-3p | 357 | 335 | O21 | | CGGGGCAGCUCAGUACAGGAU |
| hsa-miR-320b | 358 | 351 | O22 | | AAAAGCUGGGUUGAGAGGGCAA |
| hsa-miR-296-3p | 359 | 367 | O23 | | GAGGGUUGGGUGGAGGCUCUCC |
| hsa-miR-7-2-3p | 360 | 383 | O24 | | CAACAAAUCCCAGUCUACCUAA |
| hsa-miR-550a-3p | 361 | 16 | P01 | | UGUCUUACUCCCUCAGGCACAU |
| hsa-miR-380-3p | 362 | 32 | P02 | | UAUGUAAUAUGGUCCACAUCUU |
| hsa-miR-593-3p | 363 | 48 | P03 | | UGUCUCUGCUGGGGUUUCU |
| hsa-miR-1912 | 364 | 64 | P04 | | UACCCAGAGCAUGCAGUGUGAA |
| hsa-miR-493-5p | 365 | 80 | P05 | | UUGUACAUGGUAGGCUUUCAUU |
| hsa-miR-432-3p | 366 | 96 | P06 | | CUGGAUGGCUCCUCCAUGUCU |
| hsa-miR-454-5p | 367 | 112 | P07 | | ACCCUAUCAAUAUUGUCUCUGC |
| hsa-miR-936 | 368 | 128 | P08 | | ACAGUAGAGGGAGGAAUCGCAG |
| hsa-miR-30a-3p | 369 | 144 | P09 | | CUUUCAGUCGGAUGUUUGCAGC |
| hsa-let-7g-3p | 370 | 160 | P10 | | CUGUACAGGCCACUGCCUUGC |
| hsa-miR-214-5p | 371 | 176 | P11 | | UGCCUGUCUACACUUGCUGUGC |
| hsa-miR-183-3p | 372 | 192 | P12 | | GUGAAUUACCGAAGGGCCAUAA |
| hsa-miR-1179 | 373 | 208 | P13 | | AAGCAUUCUUUCAUUGGUUGG |
| hsa-miR-562 | 374 | 224 | P14 | | AAAGUAGCUGUACCAUUUGC |
| hsa-miR-579-3p | 375 | 240 | P15 | | UUCAUUUGGUAUAAACCGCGAUU |
| hsa-miR-590-3p | 376 | 256 | P16 | | UAAUUUUAUGUAUAAGCUAGU |
| hsa-miR-130a-5p | 377 | 272 | P17 | | UUCACAUUGUGCUACUGUCUGC |
| hsa-miR-563 | 378 | 288 | P18 | | AGGUUGACAUACGUUUCCC |
| hsa-miR-200a-5p | 379 | 304 | P19 | | CAUCUUACCGGACAGUGCUGGA |
| hsa-miR-483-5p | 380 | 320 | P20 | | AAGACGGGAGGAAAGAAGGGAG |
| hsa-miR-15a-3p | 381 | 336 | P21 | | CAGGCCAUAUUGUGCUGCCUCA |
| hsa-miR-944 | 382 | 352 | P22 | | AAAUUAUUGUACAUCGGAUGAG |
| hsa-miR-92a-2-5p | 383 | 368 | P23 | | GGGUGGGGAUUUGUUGCAUUAC |
| hsa-miR-548n | 384 | 384 | P24 | | CAAAAGUAAUUGUGGAUUUUGU |
|  |  |  |  | |  |
|  | | | | | |
