# Supplementary material for: Exosomal miRNAs as circulating biomarkers for prediction of development of haematogenous metastasis after surgery for stage II/III gastric cancer
Source: J Cell Mol Med. 2020 May 8;24(11):6220–32. doi: 10.1111/jcmm.15253 (PMC7294143; doi:10.1111/jcmm.15253)
Supplement: Supplementary file 4 — Table S3 [file JCMM-24-6220-s004.docx]

Supplementary Table 3. Average fold changes of miRNAs in metastatic patients

comparing with non-metastatic controls.

| **Targets** | **Normalization to miR-16-5p** | | | |
| --- | --- | --- | --- | --- |
| **Upregulation** | **Pair 1** | **Pair 2** | **Pair 3** | **Average** |
| hsa-let-7a-5p | 6.690444 | 1.492452 | 1.21891 | 3.133936 |
| hsa-let-7c-5p | 17.10818 | 1.722185 | 1.978541 | 6.9363 |
| hsa-let-7d-5p | 9.719371 | 2.02976 | 1.813886 | 4.521006 |
| hsa-let-7e-5p | 6.335696 | 1.253737 | 3.267635 | 3.619023 |
| hsa-let-7f-5p | 20.7834 | 1.519259 | 1.447557 | 7.91674 |
| hsa-let-7g-5p | 3.284298 | 1.327038 | 1.354499 | 1.988612 |
| hsa-miR-130b-3p | 3.462704 | 2.175399 | 1.915633 | 2.517912 |
| hsa-miR-139-3p | 3.337696 | 9.098707 | 4.863152 | 5.766518 |
| hsa-miR-144-5p | 8.182693 | 2.238804 | 3.545816 | 4.655771 |
| hsa-miR-16-2-3p | 6.754906 | 1.019736 | 1.403258 | 3.0593 |
| hsa-miR-18a-5p | 4.009149 | 1.419925 | 1.046196 | 2.158423 |
| hsa-miR-181b-5p | 78.16365 | 2.538438 | 1.236042 | 27.31271 |
| hsa-miR-183-5p | 9.499417 | 3.971844 | 1.275275 | 4.915512 |
| hsa-miR-186-5p | 10.61589 | 1.008571 | 2.377125 | 4.667197 |
| hsa-miR-20b-5p | 207.2298 | 1.005319 | 1.736099 | 69.99042 |
| hsa-miR-24-3p | 2.710382 | 1.129317 | 1.388068 | 1.742589 |
| hsa-miR-320a | 1.813249 | 1.921385 | 1.249943 | 1.661526 |
| hsa-miR-323a-3p | 1.891641 | 65.75732 | 95.76398 | 54.47098 |
| hsa-miR-331-3p | 9.464793 | 1.607291 | 87.86168 | 32.97792 |
| hsa-miR-335-3p | 2.183695 | 10.34185 | 291.1633 | 101.2296 |
| hsa-miR-361-3p | 3.302641 | 61.38003 | 1.758184 | 22.14695 |
| hsa-miR-374a-5p | 3.620535 | 1.02018 | 1.246923 | 1.962546 |
| hsa-miR-376c-3p | 10.66771 | 2.028619 | 1.030538 | 4.575623 |
| hsa-miR-379-5p | 303.5642 | 32.48202 | 47.4405 | 127.8289 |
| hsa-miR-410-3p | 792.457 | 49.58533 | 121.3466 | 321.1297 |
| hsa-miR-454-3p | 4.98075 | 1.1847 | 2.246171 | 2.803874 |
| hsa-miR-454-5p | 3.649639 | 2.66609 | 1.95826 | 2.757996 |
| hsa-miR-495-3p | 15.50599 | 56.83539 | 1.851719 | 24.73103 |
| hsa-miR-532-5p | 7.268008 | 1.331655 | 1.799623 | 3.466429 |
| hsa-miR-590-5p | 4.749465 | 1.175045 | 1.011882 | 2.312131 |
| hsa-miR-654-3p | 5.446656 | 46.59359 | 1.727005 | 17.92242 |
| hsa-miR-766-3p | 4.86408 | 1.312963 | 2.22758 | 2.801541 |
| hsa-miR-9-5p | 81.94068 | 2.523142 | 1.430328 | 28.63138 |
| hsa-miR-92a-3p | 1.990878 | 1.059127 | 1.219291 | 1.423099 |
| hsa-miR-98-5p | 13.57743 | 1.548987 | 2.492295 | 5.872902 |
| hsa-miR-99b-5p | 8.627287 | 1.157512 | 1.68571 | 3.823503 |
|  |  |  |  |  |
| **Downregulation** | **Pair 1** | **Pair 2** | **Pair 3** | **Average** |
| hsa-miR-18a-3p | 0.947917 | 0.897739 | 0.357905 | 0.73452 |
| hsa-miR-450a-5p | 0.8796 | 0.312145 | 0.357546 | 0.516431 |
| hsa-miR-450b-5p | 0.009089 | 0.004279 | 0.005563 | 0.00631 |
| hsa-miR-505-5p | 0.329721 | 0.006433 | 0.086647 | 0.140934 |
| hsa-miR-589-5p | 0.085555 | 0.34656 | 0.293937 | 0.242017 |
| hsa-miR-603 | 0.265275 | 0.124675 | 0.170483 | 0.186811 |
| hsa-miR-934 | 0.590063 | 0.203668 | 0.245178 | 0.346303 |

| **Targets** | **Normalization to miR-93-5p** | | | |
| --- | --- | --- | --- | --- |
| **Upregulation** | **Pair 1** | **Pair 2** | **Pair 3** | **Average** |
| hsa-let-7c-5p | 2.73435 | 3.689302 | 1.283861 | 2.569171 |
| hsa-let-7d-5p | 1.553419 | 4.348196 | 1.177018 | 2.359544 |
| hsa-let-7e-5p | 1.012616 | 2.685783 | 2.120345 | 1.939581 |
| hsa-miR-103a-3p | 2.132367 | 1.631264 | 1.512047 | 1.758559 |
| hsa-miR-107 | 1.603902 | 1.560409 | 1.021697 | 1.395336 |
| hsa-miR-1224-3p | 5.040244 | 1.117565 | 3.043876 | 3.067229 |
| hsa-miR-144-5p | 1.307816 | 4.796016 | 2.300854 | 2.801562 |
| hsa-miR-186-5p | 1.696708 | 2.160582 | 1.542499 | 1.79993 |
| hsa-miR-20b-5p | 33.12094 | 2.153617 | 1.126542 | 12.1337 |
| hsa-miR-214-3p | 93.72928 | 1.431376 | 1.225645 | 32.12877 |
| hsa-miR-29a-5p | 109.0428 | 1.709403 | 1.552405 | 37.43487 |
| hsa-miR-331-3p | 1.512731 | 3.443174 | 57.01281 | 20.65624 |
| hsa-miR-339-5p | 2.020177 | 1.059978 | 131.4062 | 44.82878 |
| hsa-miR-342-5p | 48.26955 | 1.046163 | 1.43716 | 16.91763 |
| hsa-miR-379-5p | 48.51779 | 69.5837 | 30.7838 | 49.62843 |
| hsa-miR-410-3p | 126.6561 | 106.2228 | 78.74095 | 103.8733 |
| hsa-miR-493-5p | 47.98473 | 1.837718 | 30.83724 | 26.88656 |
| hsa-miR-495-3p | 2.478278 | 121.754 | 1.201567 | 41.81129 |
| hsa-miR-502-3p | 2.792896 | 1.214082 | 2.1412 | 2.049393 |
| hsa-miR-532-5p | 1.161625 | 2.8527 | 1.167763 | 1.727362 |
| hsa-miR-95-3p | 1.274647 | 1.154651 | 45.58958 | 16.00629 |
| hsa-miR-98-5p | 2.170041 | 3.318274 | 1.617232 | 2.368516 |
| hsa-miR-99b-5p | 1.378874 | 2.479647 | 1.093845 | 1.650789 |
|  |  |  |  |  |
| **Downregulation** | **Pair 1** | **Pair 2** | **Pair 3** | **Average** |
| hsa-let-7b-3p | 0.715485 | 0.24398 | 0.520995 | 0.493487 |
| hsa-miR-106b-3p | 0.319279 | 0.315947 | 0.379149 | 0.338125 |
| hsa-miR-1244 | 0.172571 | 0.589542 | 0.196014 | 0.319376 |
| hsa-miR-125a-5p | 0.526996 | 0.422738 | 0.682292 | 0.544009 |
| hsa-miR-143-3p | 0.567524 | 0.870863 | 0.243837 | 0.560741 |
| hsa-miR-146a-5p | 0.858685 | 0.849318 | 0.645303 | 0.784435 |
| hsa-miR-146b-5p | 0.558647 | 0.921546 | 0.728506 | 0.736233 |
| hsa-miR-192-5p | 0.648657 | 0.896567 | 0.451242 | 0.665489 |
| hsa-miR-24-2-5p | 0.935556 | 0.154146 | 0.207711 | 0.432471 |
| hsa-miR-27a-3p | 0.812955 | 0.894526 | 0.506556 | 0.738012 |
| hsa-miR-340-5p | 0.783754 | 0.73012 | 0.311639 | 0.608505 |
| hsa-miR-369-3p | 0.528456 | 0.366018 | 0.495179 | 0.463218 |
| hsa-miR-375 | 0.291012 | 0.91261 | 0.804628 | 0.669417 |
| hsa-miR-431-5p | 0.280328 | 0.611131 | 0.434926 | 0.442128 |
| hsa-miR-450a-5p | 0.140584 | 0.668685 | 0.232009 | 0.347093 |
| hsa-miR-450b-5p | 0.001453 | 0.009166 | 0.00361 | 0.004743 |
| hsa-miR-483-3p | 0.88561 | 0.114556 | 0.810064 | 0.60341 |
| hsa-miR-505-5p | 0.052698 | 0.013782 | 0.056225 | 0.040902 |
| hsa-miR-548h-5p | 0.262804 | 0.239346 | 0.359874 | 0.287341 |
| hsa-miR-576-5p | 0.908115 | 0.46091 | 0.657232 | 0.675419 |
| hsa-miR-589-5p | 0.013674 | 0.742408 | 0.190734 | 0.315605 |
| hsa-miR-603 | 0.042398 | 0.267081 | 0.110625 | 0.140035 |
| hsa-miR-627-5p | 0.245041 | 0.634398 | 0.002479 | 0.293973 |
| hsa-miR-664a-3p | 0.866128 | 0.060811 | 0.401584 | 0.442841 |
| hsa-miR-93-3p | 0.427261 | 0.47543 | 0.264621 | 0.389104 |
| hsa-miR-934 | 0.094308 | 0.436301 | 0.159094 | 0.229901 |
| hsa-miR-99a-5p | 0.711963 | 0.62167 | 0.765088 | 0.699574 |
