# Supplementary material for: Exosomal miRNAs as circulating biomarkers for prediction of development of haematogenous metastasis after surgery for stage II/III gastric cancer
Source: J Cell Mol Med. 2020 May 8;24(11):6220–32. doi: 10.1111/jcmm.15253 (PMC7294143; doi:10.1111/jcmm.15253)
Supplement: Supplementary file 5 — Table S4 [file JCMM-24-6220-s005.docx]

Supplementary Table 4. Primers of miRNAs in this study.

| **miRNA (Human)** | **Sequence (Forward primer)** |
| --- | --- |
| let-7c-5p | TGAGGTAGTAGGTTGTATGGTT |
| miR-144-5p | GGATATCATCATATACTGTAAG |
| miR-379-5p | TGGTAGACTATGGAACGTAGG |
| miR-410-3p | AATATAACACAGATGGCCTGT |
| miR-98-5p | TGAGGTAGTAAGTTGTATTGTT |
| miR-505-5p | GGGAGCCAGGAAGTATTGATGT |
| miR-934 | TGTCTACTACTGGAGACACTGG |
| miR-16-5p | TAGCAGCACGTAAATATTGGCG |
| miR-93-5p | CAAAGTGCTGTTCGTGCAGGTAG |
| miR-486-3p | CGGGGCAGCTCAGTACAGGAT |
